# Phase-Dependent Adsorption of Myelin Basic Protein to Phosphatidylcholine Lipid Bilayers

**DOI:** 10.3390/membranes14010015

**Published:** 2024-01-04

**Authors:** Petra Maleš, Zlatko Brkljača, Ivo Crnolatac, Dražen Petrov, Danijela Bakarić

**Affiliations:** 1 Division for Organic Chemistry and Biochemistry, Ruđer Bošković Institute, Bijenička 54, 10000 Zagreb, Croatia; petra.males@irb.hr (P.M.); zlatko.brkljaca@selvita.com (Z.B.); ivo.crnolatac@irb.hr (I.C.); 2 Institute of Molecular Modeling and Simulation, University of Natural Resources and Life Sciences, 1180 Vienna, Austria; drazen.petrov@boku.ac.at

**Keywords:** myelin basic protein (MBP), 1,2-dipalmitoyl-*sn*-glycero-3-phosphocholine (DPPC), lipid phase-dependent hydration of MBP hydrophobic amino acids, spectroscopic (UV-Vis, FTIR, CD) and calorimetric (DSC) measurements, molecular dynamics (MD) simulations

## Abstract

The dense packing of opposite cytoplasmic surfaces of the lipid-enriched myelin membrane, responsible for the proper saltatory conduction of nerve impulses through axons, is ensured by the adhesive properties of myelin basic protein (MBP). Although preferentially interacting with negatively charged phosphatidylserine (PS) lipids, as an intrinsically disordered protein, it can easily adapt its shape to its immediate environment and thus adsorb to domains made of zwitterionic phosphatidylcholine (PC) lipids. As the molecular-level interaction pattern between MBP and PC lipid membranes suffers from scarce characterization, an experimental and computational study of multilamellar liposomes (MLVs) composed of 1,2-dipalmitoyl-*sn*-glycero-3-phosphocholine (DPPC) in the presence of bovine MBP is presented here. Calorimetric and temperature-dependent UV-Vis measurements identified DPPC pretransition temperature (*T*_p_) and calorimetric enthalpy (Δ*H*_cal_) as the physicochemical parameters most responsive to the presence of MBP. Besides suggesting an increase in β-sheet fractions of structured MBP segments as DPPC lipids undergo from the gel (20 °C) to the fluid (50 °C) phase, FTIR spectra unraveled the significant contribution of lysine (Lys) residues in the adsorption pattern, especially when DPPC is in the fluid (50 °C) phase. In addition to highlighting the importance of Lys residues in the MBP adsorption on DPPC lipid bilayer, employing salt bridges (SBs) and hydrogen bonds (HBs), MD data suggest the crucial importance of the orientation of MBP with respect to the surface of the DPPC lipid bilayer.

## 1. Introduction

Myelin is a compact, lipid-enriched, multibilayer envelope created by oligodendrocytes in the central nervous system (CNS), which wraps around the axon and ensures the proper transmission of neural impulses [1,2]. Although dominantly composed of lipids (70–80%) [3,4], the myelin sheath also contains proteins (20–30%), among which proteolipid protein (PLP) and myelin basic protein (MBP) stand out. The former is an integral and highly conserved hydrophobic protein, whereas the latter is an intrinsically unstructured positively charged protein having a charge/pI of ~+20/~11 at physiological conditions [5,6,7]. The extremely high positive charge enables MBP not just to establish electrostatic interaction with anionic phosphatidiylserine (PS) lipids present in the inner membrane leaflet but also to mediate the adhesion of opposite cytosolic leaflets, forming a compact structure with minimal cytoplasmic content [8]. In eukaryotes, MBP can be found in several isoforms [9] whose heterogeneity is further increased by their post-translation modifications in the form of deamination, phosphorylation, methylation, or the replacement of some arginine residues with citrulline [5,9,10]. Since aberrant changes in the most abundant 18.5 kDa isoform of MBP in adult humans and bovines [9,10,11] weaken MBP-membrane interactions and disrupt myelin integrity [12], MBP is addressed as a possible autoantigen in multiple sclerosis (MS) [7,13,14], a chronic immune and neurodegenerative disease [15,16] manifested through CNS inflammation, oligodendrocyte loss, or axonal degeneration.

The high net charge and relatively low hydrophobicity place MBP in the category of intrinsically disordered proteins (IDPs) whose complete lack of any secondary structure is compensated by their existence as a broad ensemble of highly dynamic and rapidly interconverted conformers [17,18,19,20]. This property makes MBP exceptionally flexible in binding to different partners [21], emphasizing negatively charged lipid membranes [22], and on these occasions, a certain portion(s) of MBP can take a defined surrounding-dictated secondary structure [5,10,11,13,17,23]. For instance, in an aqueous solution in the absence of lipid membranes, CD spectra revealed that MBP dominantly exists as a random coil structure [9,10,24,25,26] with ionic strength- [9,13] and pH-dependent [5,27] self-association. On the contrary, the presence of lipid membranes affects MBP secondary structure changes mainly in a charge-driven manner [8,22], which is additionally cross-linked by hydrophobic interactions [5,28,29]. In particular, using CD spectra, Keniry and Smith [24] established that the interaction of lipid bilayers composed from PS lipids, unlike those made of zwitterionic ones like phosphatidylcholine (PC), drives a conformational change in MBP. Moreover, not only does MBP acquire an ordered secondary structure by binding to PS lipid bilayers, in contrast to the practically negligible changes caused by binding to PC lipid bilayers, but also the binding of MBP to PS lipid bilayers is enhanced with an increase in its concentration (binding described by a sigmoidal curve), while as a result of binding to PC lipid bilayers, saturation occurs quickly (binding described by a hyperbolic curve) [5,30].

Although FTIR spectroscopy is extremely sensitive in detecting the secondary structure of proteins [14,31], the analysis of FTIR spectra of proteins in the IDP category, such as of MBP, is far from straightforward [17,32,33]. Mainly focusing on the spectral regions for secondary structure determination, the most useful vibrational bands are Amide I (1700–1600 cm^−1^), arising from the stretching of carbonyl groups (νC=O), along with Amide II (1570–1470 cm^−1^) and Amide III (1350–1250 cm^−1^) bands, originating from bending of amino moiety (δNH) and stretching of C–N bond (νCN) with different contributions [34,35]. Although different secondary structures leave their signatures at slightly different maxima in Amide I spectral region, their extensive overlap, especially for the α-helices (1660–1640 cm^−1^) and random coils (1650–1640 cm^−1^) [36], and intrinsic broadness of the particular signal make it difficult to unambiguously determine the number of constituent bands beneath the observed envelope and especially their assignment [37,38]. However, in a generally described as structural and microscopic study, Raasakka et al. [2] managed to identify a slight increase in α-helical content in MBP during its binding to PS-lipid membranes. More specifically, the neutralization of MBP upon the electrostatic interaction of the PS-lipid membrane and MBP opens the path for its folding, i.e., secondary structure change, before its insertion into the membrane [2,8,39].

Despite the preferential electrostatic interaction with PS lipids, a considerable number of studies highlight the importance of hydrophobic interactions upon MBP adsorption as the latter favors domain boundaries in model myelin membranes [28,40]. In this context, lipids in the myelin inner membrane leaflet, such as PC, must be considered as well. Besides knowing that MBP remains almost entirely at the lipid/water interface when adsorbed on a PC-lipid bilayer [8,29] and that it drives PC-based liposome aggregation [5], the attempts to build even a qualitative picture of their interaction at the molecular level are rather scarce. In one of the rare examples of characterizing the adsorption of post-translationally modified MBP on a PC-lipid membrane surface using MD simulations, Polverini et al. showed the dependence of the secondary structure of individual MBP segments on the immediate membrane or water environment [41]. In order to gain a better insight into how rather discrete environmental changes such as the lipid bilayer phase affect the MBP adsorption pattern on PC-lipid bilayers, the impact of which remains elusive [5], we examined the molecular properties of an 18.5 kDa isoform of bovine MBP adsorbed on 1,2-dipalmitoyl-*sn*-glycero-3-phosphocholine (DPPC) surface, when the latter is found either in gel (L_β′_) or fluid (L_α_) phase, using thermotropic, spectroscopic, and modelling methods. The obtained results helped us to establish how the change in lateral interactions of lipids, which is easiest to determine when the latter are in the L_β′_ (stronger in L_β′_) or L_α_ (weaker in L_α_) phase, contributes to the adhesion of MBP on the zwitterionic surface. Furthermore, they indicated the importance of forming a salt bridge between MBP and lipids, which is reflected in the way the lipid phase change is intertwined with the solvation of positively charged residues, primarily those of lysine (Lys). Nevertheless, this paper is the introduction of comprehensive studies in which thermoanalytical, spectroscopic, and computational techniques are used to shed light on the influence of lipid membrane interfaces of different composition and phase on the physicochemical properties of the 18.5 kDa isoform of bovine MBP.

## 2. Experimental Section

1,2-Dipalmitoyl-*sn*-glycero-3-phosphocholine (DPPC; white powder, Avanti Polar Lipids, ≥99%, Alabaster, AL, USA) was dissolved in chloroform (CHCl_3_; colorless liquid, Carlo Erba, p.a., Emmendingen, Germany) in order to obtain a stock solution of *γ*(DPPC) = 10 mg mL^−1^. A volume of 2.5 mL of the latter was added in flasks and the films of DPPC were produced by removing the chloroform on a rotary evaporator, followed by drying the films under an Ar stream. The 18.5 kDa isoform of bovine myelin basic protein (MBP; lyophilized delicate white powder, Sigma-Aldrich, ≥90%, Darmstadt, Germany) was dissolved in NaCl_(aq)_ (Kemika, p.a., Zagreb, Croatia) of *I*(NaCl) = 100 mM (pH~6) and the obtained concentration was *γ*(MBP) = 0.654 mg mL^−1^. When preparing the suspensions of DPPC in the absence/presence of MBP, DPPC films in individual flasks were hydrated with 5 mL of NaCl_(aq)_/5 mL of MBP in NaCl_(aq)_ in order to achieve a molar ratio of lipid/protein = 200:1 [42] in the latter. The hydration of DPPC films was followed by a three-step cycle (vortex–hot bath (70 °C)–ice bath (4 °C)) repeated multiple times until homogeneously milky-white suspensions were obtained. Although the used bath temperatures (higher than physiological) may seem harsh (they are necessary in the preparation of DPPC MLVs), due to the inherent absence of a steady secondary structure, temperature-induced protein denaturation should not be an obstacle [43,44,45,46,47]. Every step took 2 min and the whole procedure was performed in three to five cycles. In the obtained multilamellar liposome (MLV) suspensions, the mass concentration of DPPC was *γ*(DPPC) = 5 mg mL^−1^ and was diluted if needed. In the continuation of the text, MBP in NaCl_(aq)_, DPPC in NaCl_(aq)_, and MBP + DPPC in NaCl_(aq)_ will be denoted as MBP, DPPC, and MBP + DPPC, respectively.

### 2.1. DLS and Confocal Microscopy: Measurements and Data Analysis

The component size distribution was established with dynamic light scattering using a photon correlation spectrophotometer equipped with a 532 nm (green) laser (Zetasizer Nano ZS, Malvern Instruments, Worcestershire, UK). The average hydrodynamic diameter (*d*_h_) was specified as the value at the peak maximum of the volume size distribution. The reported results correspond to the average of six measurements at 25 °C. The data processing was carried out using the Zetasizer software 7.13 (Malvern Instruments). The measured average hydrodynamic diameters of DPPC MLV (*γ*(DPPC) = 0.05 mg mL^−1^, *c*(DPPC) = 67.7 μM), and MBP + DPPC MLV (*γ*(MBP) = 6.2 μg mL^−1^, *c*(MBP) = 0.3 μM + *γ*(DPPC) = 0.05 mg mL^−1^, *c*(DPPC) = 67.7 μM) were in the range 400 nm ≤ *d*_h_ ≤ 800 nm and 1200 nm ≤ *d*_h_ ≤ 1800 nm, respectively (data displayed in Supporting Information, Figure S1). The solution of MBP in NaCl_(aq)_ was measured as well (*γ*(MBP) = 0.05 mg mL^−1^, *c*(MBP) = 2.7 μM), and the size of particles on the light path were found to be in the range 500 nm ≤ *d*_h_ ≤ 800 nm (Figure S1). Along with the possibility that MBP expectedly exists as a monomer [11], the reports on MBP dimer [12] and multimer formation [48,49] in aqueous solution at this concentration suggest that, in this study, MBP very likely formed aggregates.

Confocal microscope imaging was performed on a 28 Leica TCS SP8 laser scanning confocal microscope equipped with a white light laser, using a 63× (N.A. = 1.4) oil immersion objective in transmission. The images of MBP (*γ*(MBP) = 5 mg mL^−1^, *c*(MBP) = 0.3 mM), DPPC (*γ*(DPPC) = 5 mg mL^−1^, *c*(DPPC) = 6.8 mM), and MBP + DPPC (*γ*(MBP) = 0.6 mg mL^−1^, *c*(MBP) = 33.8 μM + *γ*(DPPC) = 5 mg mL^−1^, *c*(DPPC) = 6.8 mM) are presented in the Supporting Information as Figure S2.

### 2.2. Differential Scanning Calorimetry: Measurements and Data Analysis

DSC measurements of MBP (*γ*(MBP) = 5 mg mL^−1^, *c*(MBP) = 0.3 mM) and MBP + DPPC (*γ*(MBP) = 0.6 mg mL^−1^, *c*(MBP) = 33.8 μM + *γ*(DPPC) = 5 mg mL^−1^, *c*(DPPC) = 6.8 mM) were conducted on a microcalorimeter Nano-DSC, TA Instruments (New Castle, DE, USA). After 10 min of degassing, the suspensions and MBP in NaCl_(aq)_ (filled in the cell volume of 300 μL) were heated up/cooled down in two cycles in a temperature range of 20–60 °C at a heating rate of 1 °C min^−1^ with NaCl_(aq)_ solution used as a reference. A reference (NaCl_(aq)_) scan was obtained by applying the same heating/cooling regime and the same heating rate but in a temperature interval of 10–90 °C. DSC curves of suspensions were taken two times, whereas those of a reference solution were taken once. Regarding DPPC (*γ*(DPPC) = 5 mg mL^−1^, *c*(DPPC) = 6.8 mM), the data were taken from a previous study [50].

The collected DSC curves of NaCl_(aq)_ were subtracted from the corresponding DSC curves of MBP, DPPC, and MBP + DPPC, respectively (TA Instruments Nano Analyze software package version 3.11.0), and were further analyzed in a temperature range of 30–52 °C, in which the pretransition (ripple phase formation [51] with maximum at *T*_p_) and the main phase transition (lipid melting [52] with maximum at *T*_m_) of DPPC are observed, as well as a certain endothermic event of MBP (*T*_x_), the origin of which is not yet sufficiently clarified. After manual (two-point) baseline subtraction phase transitions, the phase transition temperatures of DPPC (*T*_p_ and *T*_m_) were obtained by determining the onset (*T*_p, o_ and *T*_m, o_) and curve maximum (*T*_p, m_ and *T*_m, m_) of DPPC and MBP + DPPC of the second heating run curve, while the *T*_x_ of MBP was determined from the maximum only (*T*_x, m_). The calorimetric enthalpies (Δ*H*_cal_) of explored systems are determined by integrating the area under the thermotropic event of a DSC curve and associated entropies (Δ*S*) using the relation [53]:

(1) $$\Delta S=\frac{\Delta H_{cal}}{T_{m,\;m}}$$

in which the temperature of the maximum of the (main) thermotropic event (*T*_m, m_) expressed in K (315 K) was used.

### 2.3. Temperature-Dependent UV-Vis Spectroscopy: Spectral Acquisition and Multivariate Analysis

**Figure 1:**
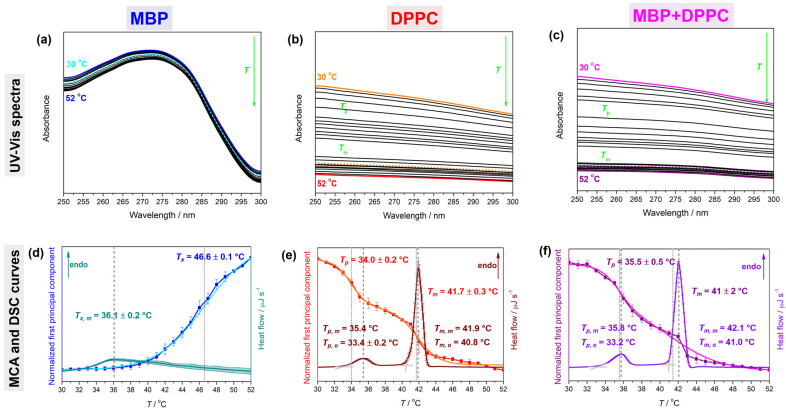
UV-Vis spectra (upper row) and DSC curves with concentration profiles of spectral projections (lower row) of UV-Vis spectra (see Section 2.3): (**a**,**d**) MBP (upper row: solid cyan/blue lines for UV-Vis spectra acquired at 30 °C/52 °C and dotted cyan line for the spectral projection obtained from MCA; lower row: dark cyan line for DSC curve, blue line for concentration profile obtained from UV-Vis spectra, and cyan line for the sigmoid fit of blue data), (**b**,**e**) DPPC (upper row: solid orange/red lines for UV-Vis spectra acquired at 30 °C/52 °C and dotted orange line for the spectral projection obtained from MCA; lower row: wine line for DSC curve, red line for concentration profile obtained from UV-Vis spectra, and orange line for the sigmoid fit of red data), and (**c**,**f**) DPPC + MBP (upper row: solid magenta/purple lines for UV-Vis spectra acquired at 30 °C/52 °C and dotted magenta line for the spectral projection obtained from MCA; lower row: violet line for DSC curve, purple line for concentration profile obtained from UV-Vis spectra, and magenta line for the sigmoid fit of purple data). Phase transition temperatures obtained from DSC data (*T*_p, o/m_, *T*_m, o/m_, and *T*_x, m_) are highlighted on graphs and labeled with dashed lines and intersection curves (DSC), while UV-Vis data (*T*_p_, *T*_m_, and *T*_x_) are designated with dotted lines.

Temperature-dependent UV-Vis spectra of MBP (*γ*(MBP) = 1 mg mL^−1^, *c*(MBP) = 54.4 μM) and MBP + DPPC (*γ*(MBP) = 0.1 mg mL^−1^, *c*(MBP) = 6.8 μM + *γ*(DPPC) = 1 mg mL^−1^, *c*(DPPC) = 1.4 mM) were collected on a UV-Vis Thermo Scientific Nanodrop 2000 spectrophotometer (Thermo Fisher Scientific, Waltham, MA, USA) in the spectral range of 250–500 nm. The samples were pipetted in covered quartz cuvettes, placed in a temperature-controlled cuvette holder, and recorded in temperature interval of 30–52 °C. The spectra of MBP and MBP + DPPC were acquired at least two times, whereas NaCl_(aq)_ spectra were used once, respectively. The data of DPPC suspension (*γ*(DPPC) = 1 mg mL^−1^, *c*(DPPC) = 1.4 mM) were taken from a previous study [50]. The obtained temperature-dependent UV-Vis spectra were smoothed (Savitzky–Golay using the polynomial of a 3rd degree through 10 points) [54] and subjected to Multivariate Curve Analysis (MCA) in the spectral range of 250–300 nm (Figure 1a–c). The choice of the latter was based on the fact that the highest temperature-dependent turbidity was observed in that range [50,55]. Using publicly available Matlab code [56], the bilinear equation system was solved:

**D** = **CS^T^** + **E** (2)

That enabled the reconstruction of our spectral data set (**D**) as a product of the concentration (**C**) and spectral (**S**) profile of the components that make up the system and within the allowed residual limit (**E**). Since one component is sufficient to describe all the temperature-dependent variability in the system, the spectra were projected onto one component and its concentration profile was analyzed in detail for all three systems: MBP, DPPC, and MBP + DPPC (Figure 1d–f). Although the concentration profile of the spectral projection of a sigmoid character was observed in all three systems, the presence of lipids introduces certain differences; for MBP itself, the concentration profile increases with temperature in a single-sigmoid fashion (single Boltzmann fit) with an inflection point at *T*_x_ = 46.6 ± 0.1 °C, (*R*^2^ = 0.999), opposing the DPPC and MBP + DPPC concentration profiles that decrease with temperature in a double-sigmoid fashion (double Boltzmann fit) with inflection points at 34.0 ± 0.2 °C and 41.7 ± 0.3 °C for DPPC (*R*^2^ = 0.995) and 35.5 ± 0.5 °C and 41 ± 2 °C for MBP + DPPC (*R*^2^ = 0.998), coinciding with *T*_p_ and *T*_m_, respectively.

### 2.4. CD Spectroscopy: Measurements and Data Analysis

CD spectra were measured on a Jasco J-815 spectrometer in quartz cells with an optical path of 0.01 cm. A volume of 40 µL of MBP (*γ*(MBP) = 1.3 mg mL^−1^, *c*(MBP) = 67.4 μM), DPPC (*γ*(DPPC) = 1 mg mL^−1^, *c*(DPPC) = 1.4 mM) and MBP + DPPC (*γ*(MBP) = 0.1 mg mL^−1^, *c*(MBP) = 6.8 μM + *γ*(DPPC) = 1 mg mL^−1^, *c*(DPPC) = 1.4 mM) and NaCl_(aq)_ was put in quartz cells placed in a temperature-controlled CD block and measured as at least two separate measurements at temperatures of 20 °C and 50 °C. The CD spectra were recorded in the wavelength range of 300–190 nm with a scanning rate of 200 nm min^−1^ and with 2 accumulations.

Subtraction of the CD spectrum of NaCl_(aq)_ from the raw CD spectra of the samples was followed by their smoothing (Savitzky–Golay; polynomial of a 3rd degree and 30 points [54]).

### 2.5. FTIR ATR: Spectral Acquisition and Signature Analysis

FTIR ATR spectra were measured on an INVENIO-S Bruker spectrometer equipped with BioATR II unit and photovoltaic LN-MCT detector using OPUS 8.5 SP1 (20200710) software. The KBr beamsplitter was set on an aperture of 1 mm and the detector was set on a scanner velocity of 15 kHz for highly sensitive measurements. The BioATR II unit was continuously purged with N_2_ gas and its temperature was regulated using a circulating water bath with a Huber Ministat 125 temperature controller. 20 μL of the samples MBP (*γ*(MBP) = 5 mg mL^−1^, *c*(MBP) = 0.3 mM), DPPC (*γ*(DPPC) = 5 mg mL^−1^, *c*(DPPC) = 6.8 mM), and MBP + DPPC (*γ*(MBP) = 0.6 mg mL^−1^, *c*(MBP) = 33.8 μM + *γ*(DPPC) = 5 mg mL^−1^, *c*(DPPC) = 6.8 mM) and NaCl_(aq)_) was placed on a small circular sample compartment (based on a dual-crystal technology) and their FTIR spectra were taken at 20 and 50 °C in order to acquire FTIR spectra of DPPC in gel (20 °C) and fluid (50 °C) phases. Three independent measurements were taken for each sample, except for NaCl_(aq)_, which was recorded once. All spectra were collected with a nominal resolution of 2 cm^−1^ and 256 or 128 scans.

The analysis of any protein spectra is complicated by the absorption of water bending (δHOH) at about 1650 cm^−1^. The resolution of superimposed species can be achieved in two ways: (i) using D_2_O [57], in which analogous normal mode (δDOD) is displaced to 1210 cm^−1^, thereby creating a window for protein signals in the Amide I region, and (ii) employing derivative spectroscopy [58], which artificially narrows the bands and improves the discrimination of the maxima of significantly overlapped bands. Despite the mentioned advantages, each of the applications has its own disadvantages; D_2_O can be utilized when it is known with certainty that it does not denature the protein [57], which is highly debatable for proteins like MBP that fall in the IDP category. Nevertheless, when applying derivative spectroscopy, one must not exaggerate when manipulating the spectra [17], which might result in the wrong assignment of spectral features; for example, some artifacts could be wrongly assigned as real and sufficiently long-lived species. However, as a careful inspection of even raw spectra can unravel certain molecular-level details, the analysis of baseline-corrected and smoothed spectral data was performed. Therefore, obtained spectra were examined in the following spectral ranges: (i) 2890–2820 cm^−1^, in which both antisymmetric (ν_as_CH_2_) and symmetric (ν_s_CH_2_) stretching of methylene groups of DPPC hydrocarbon chains appear, (ii) 1800–1480 cm^−1^, in which the band originating from carbonyl stretching of lipid glycerol backbone (νC=O(OR)) appears, along with Amide I ((νC=O(NH)),) and Amide II (δNH)) bands of MBP [35], (iii) 1490–1430 cm^−1^, which contains the signatures of DPPC methylene groups scissoring (γCH_2_) and the bending of protonated amino moiety (δNH_3_^+^) of MBP [59], (iv) 1335–1275 cm^−1^ comprising Amide III signature dominated by the bending of secondary amine (δN–H) coupled with stretching of CN (νC–N) and bending of CH (δCH) moiety of MBP [60,61], (v) 1275–1190 cm^−1^, which reveals the signatures of phosphate moieties of lipid polar headgroups (ν_as_PO_2_^−^) and bending of secondary amine (δN–H) coupled with stretching of CN moiety (νC–N) and bending of CH moiety (δCH) [60] of MBP, and (vi) 1015–935 cm^−1^ containing the signature of a choline group of lipid molecules (ν_(a)s_C–N(CH_3_)_3_^+^) [62,63]. In the selected ranges, the spectra were smoothed (Savitzky–Golay procedure using the polynomial of a 3rd degree through 10/40 points (256/128 scans) and baseline-corrected (2 points at spectral minima) [54].

To make it easier to follow the text, in Section 4 (Results and Discussion), the experimental results are presented in such a way that all data related to MBP are blue-shaded, for DPPC red-shaded, and for the mixture MBP + DPPC purple-shaded.

## 3. Molecular Dynamics Simulations

Molecular dynamics (MD) simulations were used to model MBP in contact with the DPPC membrane. The simulated systems contained a bilayer of 620 DPPC lipids, one MBP molecule (native sequence without modifications with expected protonation at pH 7, i.e., lysine and arginine +1 and glutamic acid and aspartic acid −1), and 89 sodium and 109 chloride ions (ensuring overall charge neutrality in the system), all solvated in 59629 TIP3P water molecules [64]. The initial membrane configuration with an equilibration protocol was obtained using CHARMM GUI, where the membrane was brought to 1-bar pressure and temperatures of 20 °C and 50 °C in a stepwise thermalization procedure, for each of the two studied systems, respectively. Subsequently, MBP structure was placed in close proximity to the membrane in two different orientations that rotated 180 degrees in respect to each other, with one of its structured parts (resolved by NMR, PDB code 2LUG [65]) facing away and towards the membrane (Figure S4). The initial MBP structure was obtained using AlphaFold2 [66] prediction program. After the removal of all overlapping water molecules, an additional equilibration step was performed to thermalize MBP to the desired temperature. These equilibrated systems were used as initial configurations for the production of MD simulations.

MD simulations were performed using the GROMACS 2020 simulation package [67], CHARMM36m force field [68], and a 2 fs integration step, with a simulation length of 200 ns. All bonds involving hydrogen atoms were constrained using the LINCS algorithm [69]. The pressure was kept at 1 bar using the semi-isotropic Parrinello–Rahman barostat [70] in combination with a relaxation time of 5 ps and compressibility of 4.5 × 10^−5^ bar^−1^, while the temperature was maintained at 20 °C and 50 °C using the Nose–Hoover thermostat [71,72] in conjunction with a relaxation time of 1 ps and 10 chains. Electrostatic interactions were approximated using the particle mesh Ewald (PME) method, while the Van der Waals interactions were calculated up to a 1.2 nm cutoff distance so that the forces were smoothly switched to 0 between 1 nm and the 1.2 nm cutoff distance. All analyses were performed using GROMACS analysis tools and programs, as well as custom-made Python scripts.

## 4. Results and Discussion

### 4.1. Thermotropic Properties of MBP, DPPC, and MBP + DPPC: DSC and UV-Vis Data

As evidenced by the temperature-dependent UV-Vis spectra (Figure 1a) and DSC curves (Figure 1d), the temperature rise induced a very weak endothermic event on MBP with a maximum determined from DSC/UV-Vis data at 36.1 ± 0.2 °C/46.6 ± 0.6 °C. This reversible event (Figure S3) probably stems from heat-induced structural changes within MBP (like compaction [43,46,73]), but what is intriguing is the displacement of values obtained from DSC and UV-Vis spectra for about 10 °C. There is the possibility that the absorption of chromophores in MBP [25] interferes and ultimately modulates the thermotropic event observed at about 36 °C in the DSC experiment, or simply that the lipid bilayer-based approach [50,55,74] requires certain modifications when protein solutions are explored. As the interpretation of the origin of this event is the topic of our ongoing research, it will not be further discussed in this manuscript. Following previously reported findings [50,55], in the temperature range of 30–52 °C, DPPC (Figure 1b,c,e,f) underwent a low-cooperativity pretransition (having the maximum at *T*_p_) and a high-cooperativity main phase transition (having the maximum at *T*_m_). The physicochemical basis of the mentioned phase transitions can be found elsewhere (see [51] and references therein). The phase transition temperatures of DPPC as determined from the onset _(o)_/maximum _(m)_ of the DSC curve are *T*_p_: 33.4 ± 0.2 °C/35.4 ± 0.1 °C and *T*_m_: 40.8 ± 0.1 °C/41.9 ± 0.1 °C, whereas the analogous data for MBP + DPPC are *T*_p_: 33.2 ± 0.1 °C/35.8 ± 0.1 °C and *T*_m_: 41.0 ± 0.1 °C/42.1 ± 0.1 °C. The corresponding data determined from MCA of spectral projections of temperature-dependent UV-Vis spectra are *T*_p_ = 34.0 ± 0.2 °C and *T*_m_ = 41.7 ± 0.3 °C for DPPC [50] and *T*_p_ = 35.5 ± 0.5 °C and *T*_m_ = 41 ± 2 °C for MBP + DPPC (all data are listed in Table 1).

The obtained Δ*H*_cal_/Δ*S* (Table 1) values of thermotropic events of MBP (8.3 ± 0.1 kJ mol^−1^/27.7 ± 0.2 J K^−1^ mol^−1^), DPPC (26.9 ± 0.2 kJ mol^−1^/85.4 ± 0.6 J K^−1^ mol^−1^, which matches values reported by other authors [75,76]) and MBP + DPPC (62 ± 1 kJ mol^−1^/197 ± 4 J K^−1^ mol^−1^) imply a high cooperativity of the melting of DPPC multibilayers and the thermotropic event of MBP. Although at this end, a (semi)fusion and/or MBP-assisted/induced aggregation of DPPC MLV (Figure S2 in Supporting Information) [5,9,27] might be assigned as a possible cause of the measured phenomenon, at the MBP concentrations used in the DSC experiments (as well as in the FTIR and UV-Vis measurements), the possibility that MBP molecules aggregate even in the absence of DPPC or even upon heating also has to be considered. Thus, in the continuation of the text, whenever MBP is discussed, it could stand for both MBP monomer and MBP aggregates. Ultimately, more details on this issue will be provided in our subsequent publication. Interestingly, based on the temperature values of the phase transitions determined from the DSC curves, it cannot be said that MBP introduces any significant change in the pretransition and the main phase transition, but this is not the case when considering the data obtained from the temperature-dependent UV-Vis spectra. The increase in *T*_p_ by about 1 °C and the unchanged *T*_m_ of DPPC in the presence of MBP suggest the following: (i) their interaction is localized on the lipid bilayer surface, and (ii) the presence of MBP indirectly strengthens the lateral interactions between lipids and/or significantly affects the interfacial water layer (that encompasses the region from the glycerol backbone to the choline group), and/or realizes direct dominantly electrostatic interactions with polar groups of lipid molecules, which are reflected in the undulations of the surface of the lipid bilayer. More details on the molecular-level interactions are obtainable from the spectroscopic and modeling data.

### 4.2. Spectroscopic and Molecular Properties of MBP, DPPC, and MBP + DPPC: CD, FTIR, and Modeling Data (MD Data)

#### 4.2.1. CD Spectra

**Figure 2:**
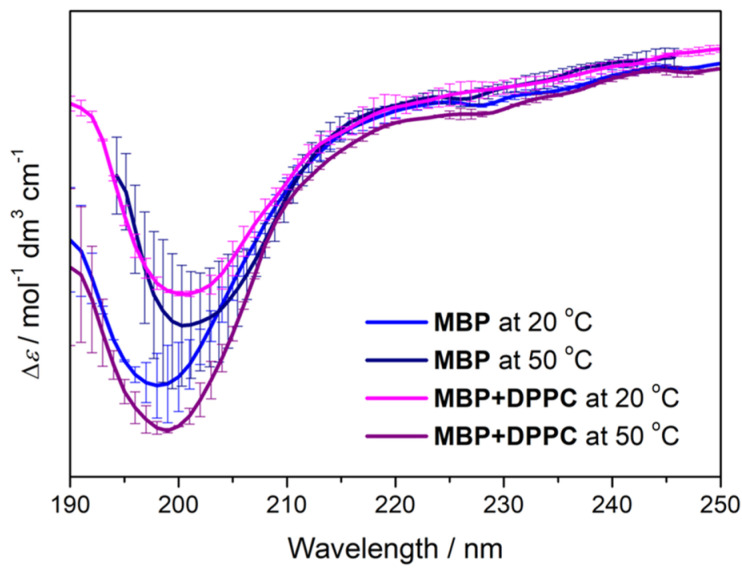
CD spectra of MBP at 20 °C (blue); MBP at 50 °C (navy); MBP + DPPC at 20 °C (magenta); MBP + DPPC at 50 °C (purple).

By producing a strong negative envelope with a maximum between 190 nm and 200 nm, the CD spectra of MBP solutions acquired at 20 °C and 50 °C (Figure 2) correspond to those characteristic of IDPs [77,78]. The displacement of the maximum and envelope broadening in the CD spectra of MBP + DPPC suspensions (both at 20 °C and 50 °C) might be related to either transient formation of ordered secondary structures and/or their redistribution within MBP when adsorbed on DPPC [79]. The induced CD effect [80,81], occurring when the intrinsically non-chiral molecule interacts with the chiral one, cannot be excluded either. Although the influence of the sample turbidity might also contribute to the overall appearance of CD spectra, especially at lower temperatures, in the systems examined here, the signal generated by MBP, both in NaCl_(aq)_ and in DPPC suspension, far exceeds the signature of the turbidity of the suspensions (Supporting Information, Figure S5).

#### 4.2.2. FTIR Spectra

**Figure 3:**
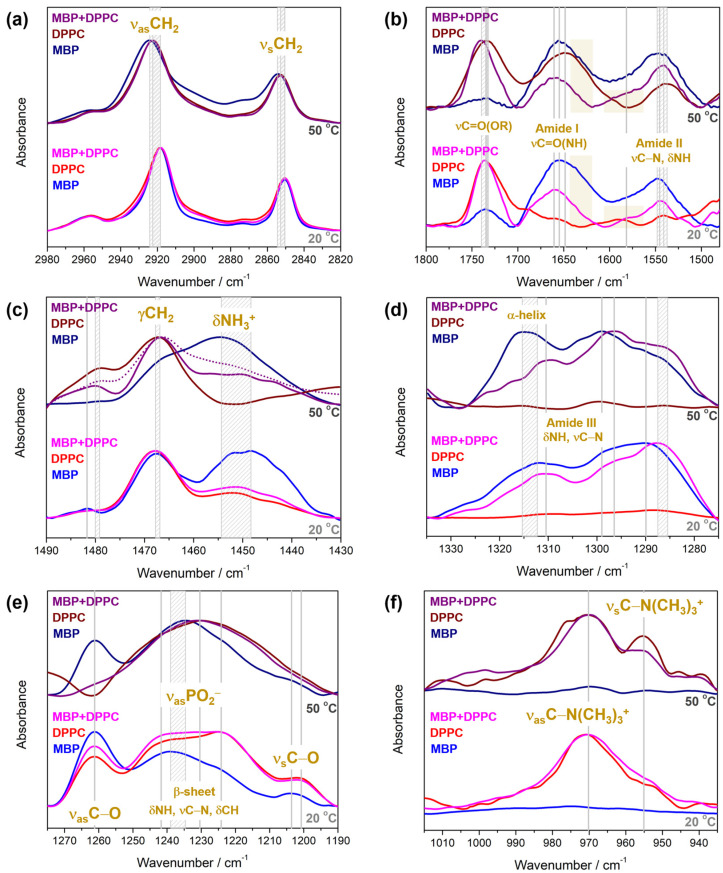
FTIR spectra of MBP, DPPC, and MBP + DPPC collected at 20 °C and 50 °C divided into the following spectral regions: (**a**) 2980–2820 cm^−1^ (ν_as_CH_2_ and ν_s_ CH_2_); (**b**) 1800–1480 cm^−1^ (((νC=O)OR, Amide I ((νC=O)NH), (ν_as_COO^−^) and Amide II (δNH)); (**c**) 1490–1430 cm^−1^ (γCH_2_ and δNH_3_^+^); (**d**) 1335–1275 cm^−1^ (Amide III (δNH)); (**e**) 1275–1190 cm^−1^ (ν_(a)s_C–O, ν_as_PO_2_^−^, MBP side-chain bands [35]); (**f**) 1015–935 cm^−1^ (ν_(a)s_C–N(CH_3_)_3_^+^). The spectra of MBP, DPPC, and MBP + DPPC at 20 °C/50 °C are labelled in blue/navy, red/wine, and magenta/purple, respectively.

In spectral region 2980–2820 cm^−1^ (Figure 3a), the bands originated from methylene group stretching appear: ν_as_ and ν_s_ at 2918 cm^−1^ and 2850 cm^−1^ at 20 °C observed in the FTIR spectra in both pure compounds (MBP, DPPC) and their mixture (MBP + DPPC) displace to higher frequencies at 50 °C: 2924 cm^−1^ (DPPC, MBP + DPPC)/2925 cm^−1^ (MBP) and 2853 cm^−1^.

After subtracting NaCl_(aq)_ spectrum, in spectral region 1800–1480 cm^−1^ (Figure 3b), MBP displays broad irregularly shaped features with three distinguished maxima that presumably originate from the stretching of carbonyl group (νC=O(OR)), Amide I band, antisymmetric stretching of deprotonated carboxylic group (ν_as_COO^−^) (1600–1575 cm^−1^ beneath yellow rectangle), and Amide II band, respectively. The maxima of νC=O and Amide II band observed at 1733 cm^−1^ (20 °C) and 1548 cm^−1^ (20 °C), respectively, display a low-frequency shift to 1733 cm^−1^ (50 °C) and 1545 cm^−1^ (50 °C) upon heating, whereas Amide I band with a maximum at 1655 cm^−1^ (20 °C) remains at the same place (1655 cm^−1^ at 50 °C). Additionally, although the heating causes an intensity gain of Amide II band, even at 50 °C Amide I band is stronger than Amide II band. A small low-frequency shift might be linked to the temperature-dependent hydration changes, which are closely related to the event observed from the DSC measurements of MBP. In broader terms, this might be interpreted as the strengthening of hydrogen bonds (HBs) formed either between MBP and water molecules or the ones entangled by HB-donating and HB-accepting moieties of MBP. In the same spectral region of DPPC at 20 °C, two distinguished bands are presumed to be the signatures of the lipid glycerol backbone: νC=O(OR) and δCHO [82], the concentration of which increases with time. As the temperature rises, the former envelope undergoes a low-frequency shift from 1736 cm^−1^ (20 °C) to 1734 cm^−1^ (50 °C) and from 1542 cm^−1^ (20 °C) to 1539 cm^−1^. (50 °C). The enhancement of weak band features in the middle of the spectral region with increasing temperature results in an envelope that displays a maximum at 1649 cm^−1^ at 50 °C, which originates from the bending of water molecules (δHOH). The general broadening of νC=O band with temperature, highlighted especially on the low-frequency side, suggests a significant increase in carbonyl groups involved in HB dominantly with water molecules. Although FTIR spectrum of MBP + DPPC is a superposition of the spectra of individual components, there are certain differences in the spectral signatures’ intensity ratios. As there is much more DPPC in the mixture than MBP, it is expected that νC=O band is the strongest at both temperatures, with maxima observed at 1736 cm^−1^ at 20 °C and 1740 cm^−1^ at 50 °C. Intriguingly, it displays a high-frequency shift upon heating, suggesting a decrease in the number of HBs with water, which could be compensated with other partners. Amide I band displays a maximum at both temperatures at the same wavenumber (1660 cm^−1^ at both 20 °C and 50 °C), and Amide II displaces from 1546 cm^−1^ (20 °C) to 1542 cm^−1^ (50 °C). The intensities of Amide I and Amide II bands are inverted upon heating in the presence of DPPC (Amide II gets stronger), which does not occur in DPPC absence. Additionally, the high-frequency band feature of Amide II band that spans the range 1620–1565 cm^−1^, very likely attributed to the (ν_as_COO^−^), is resolved better in the presence of DPPC. Interestingly, as Amide I envelope in the presence of DPPC gets narrower on the low-frequency side (1640–1600 cm^−1^, labelled with a yellow rectangle) at both temperatures, and Amide II displays an intensity enhancement along with a small-but-significant low-frequency wavenumber shift, it is reasonable to conclude that the presence of DPPC in L_α_ phase (50 °C) causes a significant increase in ordered structures, in particular in β-sheets [83].

Spectral region 1490–1430 cm^−1^ contains the signatures of both MBP and DPPC (Figure 3c). In FTIR spectrum of MBP the band at 1482 cm^−1^ detected at 20 °C disappears at 50 °C, while in FTIR spectrum of DPPC at 1479 cm^−1^, there is a band at 50 °C that does not appear at 20 °C. The corresponding band appears in FTIR spectrum of MBP + DPPC at 1480 cm^−1^ at both 20 °C and 50 °C. Further, at 20 °C, MBP displays two broad envelopes: the former is more symmetric and has a maximum at 1468 cm^−1^, whereas the latter is much broader, relatively nonsymmetric with two shallow maxima at 1452 cm^−1^ and 1449 cm^−1^, and is assigned as δNH_3_^+^. When heated up to 50 °C, these features approach each other, resulting in one broad envelope with two maxima at 1466 cm^−1^ and 1455 cm^−1^. As expected, in the examined spectral range, DPPC displays a band originating from γCH_2_ with a maximum at 1468 cm^−1^/1467 cm^−1^ at 20 °C/50 °C, along with two weak maxima at 1452 cm^−1^ and 1444 cm^−1^ at 20 °C due to the wagging progression of methylene groups (δCH_2_) that disappear upon heating [59]. Additionally, a very weak feature on the high-frequency side of γCH_2_ at 20 °C becomes more prominent at 50 °C and absorbs at 1479 cm^−1^. The FTIR spectrum of MBP + DPPC at 20 °C resembles the spectrum of pure DPPC (1468 cm^−1^, 1452 cm^−1^, 1444 cm^−1^); i.e., there are no intense band features in the range 1460–1440 cm^−1^. However, upon heating up to 50 °C, besides the band originating from γCH_2_ at 1467 cm^−1^ and the associated high-frequency shoulder at 1480 cm^−1^, there is also an intensity gain on the low-frequency side of the γCH_2_ band in the range 1460–1440 cm^−1^ (δNH_3_^+^). Although this envelope does not display as significant an intensity enhancement as in the case of pure MBP, it is evident that the temperature rise favors this band enhancement compared to the spectrum of MBP + DPPC acquired at 20 °C. Moreover, when MBP and DPPC spectra are superimposed, the resulting band is expected at higher frequencies than found in the spectrum of MBP + DPPC mixture (dotted-line spectrum), reflecting either the interaction of protonated amino group δNH_3_^+^ of Lys residue(s) in MBP with DPPC or that DPPC induces/strengthens the interaction between different (parts of) MBP molecules, both of which become prominent when DPPC is in L_α_ phase. Based on the phase-dependent spectral changes in the carbonyl stretching region (see the previous paragraph), this spectral phenomenon is taken as a confirmation that the mentioned conformational change in MBP is associated with the gel → fluid phase transition of DPPC.

In spectral range 1335–1275 cm^−1^ (Figure 3d) only MBP displays distinguished signatures, in particular of Amide III band, stemming predominantly from the bending of amino group (δNH) coupled with the stretching of C–N moiety (νC–N), making it sensitive to both the protein backbone and side chains [35]. In particular, FTIR spectra of aqueous solution of MBP display an irregular band pattern with two relatively well-defined maxima at 1312 cm^−1^ and 1290 cm^−1^ at 20 °C. As the temperature increases to 50 °C, the former band feature undergoes a high-frequency shift to 1315 cm^−1^ and exceeds the intensity of the latter one, displacing up to 1299 cm^−1^. In the presence of DPPC, the band at 1310 cm^−1^ remains at the same position upon heating (20 °C/50 °C), but the feature with a maximum at 1288 cm^−1^ displaces to 1286 cm^−1^. Further, as the low-frequency displacement is accompanied by the intensity increase in the feature with the maximum estimated to be at 1296 cm^−1^, analogously to the behavior of pure MBP, this trend is assigned as a decrease in the α-helical content of MBP in the presence of DPPC in fluid phase [60,61].

Spectral region 1275–1190 cm^−1^ (Figure 3e) unravels the signatures at 1261 cm^−1^ observed in the spectra of both pure compounds and their mixture at 20 °C: in MBP, this is the signature of coupled δNH, νC-N, and δCH vibrations (β-sheet signatures [60,61]), whereas in DPPC, the band originates from νasPO2-, both HB and non-HB. However, upon heating, this band remains only in FTIR spectrum of MBP and disappears/decreases in intensity in DPPC and MBP + DPPC spectra (or is simply too weak to be detected). A broad feature with a maximum at 1239 cm^−1^ at 20 °C displaced to 1235 cm^−1^ at 50 °C, whereas a weak band at 1204 cm^−1^ retained its position upon heating. FTIR spectra of DPPC and MBP + DPPC mixture resemble each other in the way that they display a merging of the bands at 1242 cm^−1^ and 1224 cm^−1^ at 20 °C to 1230 cm^−1^ at 50 °C, suggesting that the signals originated from HB and non-HB phosphate are superimposed and merged beneath one broad band feature. The band at 1201 cm^−1^ disappears upon heating.

Finally, the spectral region 1015–395 cm^−1^ unveils the signature of choline moiety (ν_(a)s_C–N(CH_3_)_3_^+^) of DPPC molecules that remains unchanged as the temperature rises (Figure 3f): at both 20 °C and 50 °C, the signals appear at 970 cm^−1^ (ν_as_) and 955 cm^−1^ (ν_s_) 20 °C. Although due to the retention of the position of the band, it can be assumed that no significant interaction occurs between MBP and the choline group of DPPC lipids, the narrower signals of the choline group at 50 °C in the presence of MBP may be affected by the possibly constrained rotation of methyl groups in the presence of adsorbed MBP [63].

#### 4.2.3. Modeling Data

**Figure 4:**
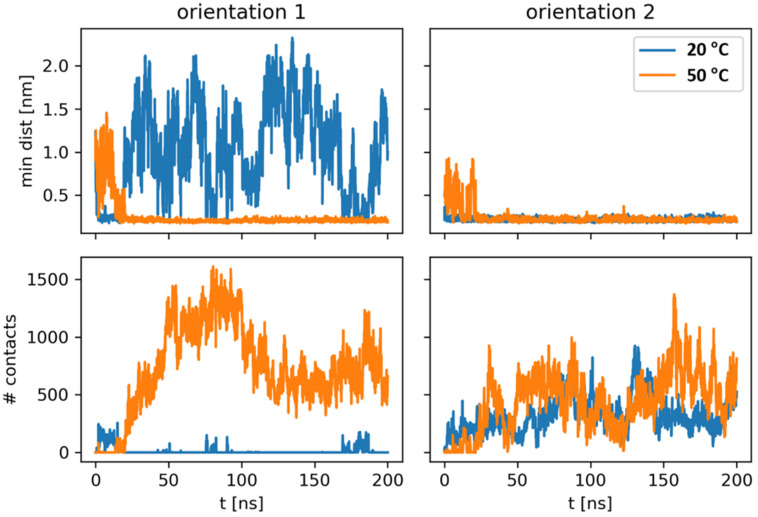
Interactions between MBP and DPPC. MBP tends to bind the membrane at both simulated temperatures, although orientation 1 shows strong difference in binding depending on the simulated temperature. Minimal distance (upper row) and number of contacts (lower row) are shown as functions of simulated time in blue (lower temperature) and orange (higher temperature).

To closely examine how MBP and the DPPC membrane interact with each other at the molecular level, MD simulations were performed at 20 °C and 50 °C (Figure 4).

Before discussing interactions between the protein and the lipid bilayer, the membrane itself was analyzed. As expected, DPPC displayed different behavior at lower and higher temperatures. In agreement with experimental data, upon an increase in temperature from 20 °C to 50 °C, DPPC showed an increase in area per lipid (from 0.5 nm^2^ to 0.6 nm^2^) and a decrease in membrane thickness (4.8 nm to 4.0 nm) (Figure S6). This shows that the models describe the lipid bilayer in the correct phase, the gel and the fluid, respectively. Additionally, this is in accordance with previous modelling work [41,55].

**Figure 5:**
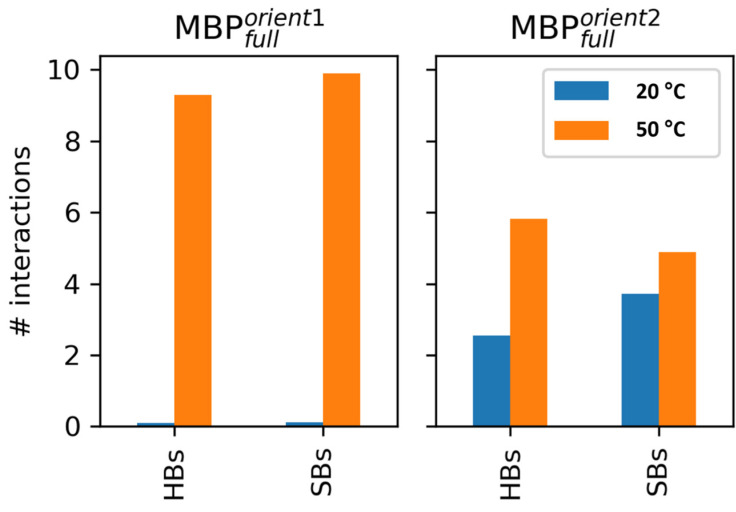
Hydrogen bonds (HBs) and salt bridges (SBs) between MBP and DPPC. This analysis reveals that the MBP interacts with the membrane both via HBs and SBs, where the total number of such interactions is greater at the higher simulated temperature. Orientation 1 and 2 are presented in the left and the right panel, while lower and higher temperatures in blue and orange.

All systems were simulated in the same fashion, such that MBP was placed in two different orientations in proximity to the membrane, and the system was let to evolve. In orientation 1, which features the structured part of the protein facing away from the membrane (Figure S4, left), MBP strongly interacted with the membrane at the higher temperature while only sporadically forming a few weak contacts with the bilayer at the lower temperature (Figure 4, left). Most of the contacts were formed via HBs and salt bridges (SBs) (approximately 9 and 10 on average throughout the simulation, Figure 5). Additionally, the phosphate groups predominantly formed contacts with MBP in this orientation at 50 °C (Figure S7), highlighting the importance of their interaction with positively charged (lysine (Lys) and arginine (Arg)) residues of the protein. In orientation 2, in which the structured part faces towards the membrane (Figure S4, right), MBP at both temperatures stayed in contact with the membrane throughout the simulation (Figure 4, right). In both cases, the number of contacts between the protein and the membrane fluctuated similarly but reached smaller values compared to orientation 1 at 50 °C. Interestingly, although the overall number of contacts is similar, the number of HBs and SBs is smaller in the case of the lower-temperature system. Additionally, it was observed that, in general, MBP created contacts predominantly with the phosphate groups of the membrane at the higher temperature, whereas phosphate and choline groups contributed equally to interactions in the gel phase (Figure S7).

**Figure 6:**
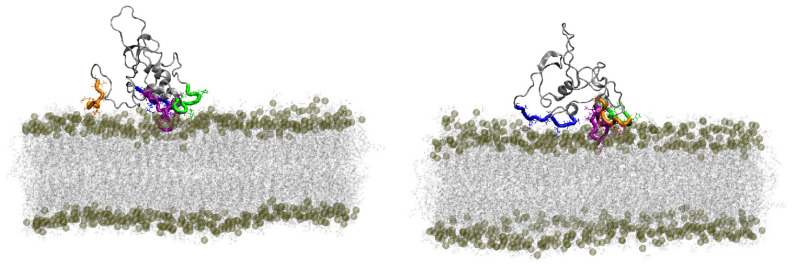
Two views at the structure of MBP (orientation 1, *T* = 50 °C) possessing most contacts/interactions with DPPC during its respective simulation. Phosphorous atoms belonging to the lipid headgroups are shown in transparent yellow, with the remainder of the bilayer given in gray. Protein regions interacting with the DPPC bilayer are shown in orange (residues 1 to 8), blue (residues 45 to 55), green (residues 136 to 144), and purple (residues 153 to 169), with the last two belonging to the extended C-terminus region.

As aforementioned, the positively charged residues, which are abundant in MBP, are seemingly the driving force behind the non-covalent binding of MBP to the lipid bilayer. However, it is a priori unclear whether certain regions of MBP are more favored to interact with the lipid headgroups. Bearing this in mind, a detailed analysis of hydrogen bond interactions and contacts between DPPC and all protein residues was performed (Tables S1 and S2) for all four simulated scenarios. The major interacting residues, for the three cases in which MBP is in contact with the lipid bilayer, are given in Table 2. The performed analysis shows that positively charged Arg and Lys residues indeed dominate the interaction landscape of MBP-lipid bilayer systems, with different regions of the protein being in position to favorably interact with the membrane. However, this analysis also points to a tentative possibility that, regardless of MBP being potentially a highly promiscuous protein both structure- and interaction-wise, certain parts of MBP are seemingly more likely to initiate the investigated process, with the extended C-terminus (considering all available data, approximately the region encompassing residues 120 to 169) being the region which consistently interacts with the lipid bilayer, regardless of the initial orientation (Table 2, Tables S1 and S2, Figure 6, and Figure S8).

The experimental data show that temperature and contact with the membrane affect the secondary structure of MBP. We performed principal component analysis, based on the Cα atoms of MBP’s backbone (Figure S9), whereby it was revealed that MBP which is not in contact with the lipid bilayer (orientation 1, *T* = 20 °C scenario; consult Figure 4) possesses significantly larger conformational space compared to the remaining (in contact) cases. Moreover, it was found that the flexibility of MBP greatly depends on the initial orientation of the protein and the specific interactions it forms with the lipid bilayer (compare the conformational phase space of orientation 1 and orientation 2 at 50 °C, Figure S9). It is also worth mentioning that the lipid bilayer is seemingly not (globally) affected by the presence of MBP, as evident from the calculated deuterium order parameters of *sn*1 and *sn*2 acyl chains belonging to DPPC molecules (see Figure S10). However, one has to take into account that (i) MBP is an intrinsically disordered protein (IDP), and the predicted 3D structure (used as the initial structure for simulations) represents only one of the possible conformations that the protein might take, and (ii) the time scales needed for monitoring structural changes in MBP in a statistically robust and meaningful manner are currently not tractable by MD simulations. Nevertheless, this initial model structure of MBP represents a rather extended unfolded conformation (expected for an IDP), which, as showcased, allowed us to examine the manner in which different parts of the structure behave when in contact with the membrane (or in proximity). This simulated process relates to the early events of MBP binding to the membrane, where the importance of SBs, especially of positively charged amino acids of MBP, was revealed from simulated data. Moreover, the simulations show that the orientation of MBP, i.e., the surface encountering the membrane, may be an essential factor in the strength and type of interactions that the protein forms with the bilayer. Finally, simulations also tentatively point to a certain MBP region, namely the extended C-terminus, as potentially playing an important role in the investigated phenomenon.

Considering all the experimental and computational data, the surface of DPPC MLVs can be understood as a scaffold for MBP adsorption whose structural features are reflected in the way MBP adheres. As FTIR data reveal that MBP adheres differently when DPPC MLVs are in the gel (20 °C) and fluid (50 °C) phases, MBP orientation 1 seems to be dominant during its adsorption on the surface of DPPC MLVs (MD data). Apart from indicating that adhesion to fluid-phase bilayers might favor an increase in the (antiparallel) β-sheet fraction (according to FTIR data at 50 °C), it appears that the latter directly encompasses Arg (MD data) and Lys residues (FTIR and MD data), very likely by employing SBs between guanidinium/–NH_3_^+^ moieties (MBP) and phosphate groups (DPPC). Ultimately, the assumption that the interaction of scattered Arg and Lys residues of MBP, when DPPC is in the fluid phase, induces the helical-to-sheet transition of MBP structured parts highlights the critical importance of MBP’s orientation towards the lipid bilayer surface, implicating the possible favoring of a particular orientation depending on the charge of the lipid membrane to which MBP adheres. 

## 5. Conclusions

In this work, the interaction pattern of the 18.5 kDa isoform of bovine MBP with MLVs composed of DPPC lipids using experimental and computational techniques was explored. The change in the thermotropic properties of MLVs of DPPC in the presence of MBP manifested through the increase in *T*_p_ of DPPC MLVs and Δ*H*_cal_ during DPPC melting is a consequence of not just MBP adsorption but probably also of MBP, MLVs, and MBP-assisted MLV aggregation. The only thing that can be safely concluded from the CD spectra is that, regardless of the absence/presence of DPPC MLVs, MBP remains, on average, equally unstructured. FTIR spectra of MBP in the presence of DPPC suggest not just an increase in (antiparallel) β-sheet fraction at 50 °C but also a direct involvement of Lys amino acid residues when MBP is adsorbed on DPPC, especially when the latter is in L_α_ phase. Besides supporting FTIR results regarding the role of Lys and Arg residues in forming SBs with phosphate moieties of DPPC in the fluid phase, MD data reveal that the orientation of MBP with respect to the membrane surface is critical for its adhesion fashion. Ultimately, the contribution of Lys and Arg residues in interaction with DPPC implies that the anchoring of MBP through SBs might induce local transient changes in the secondary structure, the effect of which resembles those in antimicrobial and cell-penetrating peptides that are also enriched in Lys and Arg residues.

## Figures and Tables

**Table 1:** Phase transition temperatures (*T*_pt_) and Δ*H*_cal_ and Δ*S* for MBP unspecified event; DPPC: pretransition and main phase transition; MBP + DPPC: pretransition and main phase transition. *T*_pt_ are determined from (i) onset (_o_) and/or maximum (_m_) of DSC curves of MBP (*T*_x, m_), DPPC (*T*_p, o/m_, *T*_m, o/m_), and MBP + DPPC (*T*_p, o/m_, *T*_m, o/m_) and (ii) from the inflection points of spectral projections of temperature-dependent UV-Vis spectra of MBP (*T*_x_), DPPC (*T*_p_, *T*_m_), and MBP + DPPC (*T*_p_, *T*_m_).

System	*T*_pt_ ^a^				Δ*H*_cal_ ^c^	Δ*S* ^d,e^
	DSC ^b^		UV-Vis			
	*T*_p, o/m_	*T*_m, o/m_	*T*_p_	*T*_m_		
MBP ^f^	36.1 ± 0.2		46.6 ± 0.1		8.6 ± 0.1	27.7 ± 0.2
DPPC	33.4 ± 0.2/35.4	40.8/41.9	34.0 ± 0.2	41.7 ± 0.3	26.9 ± 0.2	85.4 ± 0.6
MBP + DPPC	33.2/35.8	41.0/42.1	35.5 ± 0.5	41 ± 2	62 ± 1	197 ± 4

^a^ in °C; ^b^ if not specified otherwise, the uncertainty in DSC measurements is ± 0.1 °C; ^c^ in kJ mol^−1^; ^d^ in J K^−1^ mol^−1^; ^e^ determined using *T* = 315 K (42 °C); ^f^ for MBP, the unspecified event is characterized from DSC curve maximum and labeled as *T*_x, m_.

**Table 2:** Protein residues showcasing most dominant interactions with the lipid bilayer, with both average number (#) of HBs and of contacts in the last 150 ns of their respective MD simulations reported.

Orientation 2, *T* = 20 °C			Orientation 1, *T* = 50 °C			Orientation 2, *T* = 50 °C		
Residue	Average # of HBs	Average # of Contacts	Residue	Average # of HBs	Average # of Contacts	Residue	Average # of HBs	Average # of Contacts
**129ARG**	1.0	177.1	158ARG	2.7	247.4	129ARG	1.9	202.8
**51LYS**	0.7	92.1	161ARG	2.2	329.3	169ARG	1.1	113.4
**4LYS**	0.1	21.2	162SER	1.0	93.3	168ARG	0.9	122.9
**1ALA**	0.1	24.6	160SER	0.8	211.1	112ARG	0.7	62.4
**128GLY**	0.1	45.1	169ARG	0.8	127.9	104LYS	0.4	50.3
**134LYS**	0.1	14.2	47ARG	0.8	125.8	134LYS	0.4	53.9
**2ALA**	0.1	10.8	168ARG	0.5	81.7	161ARG	0.3	27.7
**169ARG**	0.1	15.6	159ASP	0.5	143.8	109SER	0.2	25.6
**131SER**	0.1	14.9	5ARG	0.4	30.9	138LYS	0.2	26.8
**168ARG**	0.1	5.8	141LYS	0.3	59.2	128GLY	0.1	44.4

## Data Availability

Data is contained within the paper and Supplementary Materials.

## Supplementary Materials

The following supporting information can be downloaded at: https://www.mdpi.com/article/10.3390/membranes14010015/s1, Figure S1: DLS data of: (a) MBP; (b) DPPC; (c) MBP + DPPC in NaCl_(aq)_ obtained at 25 °C; Figure S2: Confocal microscope images of: DPPC (upper row: a–c) and MBP + DPPC (lower row: d–f) in transmission; Figure S3: DSC curves (raw data) of MBP in NaCl_(aq)_ obtained from the first (gray) and second (dark cyan) heating runs. Dashed lines designated estimated maximum of very weak thermotropic event; Figure S4: Equilibrated structures used as initial configurations for production simulations at 20 °C. MBP orientation 1 and orientation 2 (left and right, respectively); Figure S5: Mean smoothed CD spectra of MBP, DPPC and MBP + DPPC measured at 20 °C and 50 °C; Figure S6: Membrane area per lipid (APL; nm^2^) and thickness (nm) at 20 °C and 50 °C. As expected, with increased temperature, the area per lipid increases and thickness decreases. The properties are consistent in simulation of MBP in different initial orientation; Figure S7: Type of interactions between MBP and DPPC. At higher temperature MBP forms contacts predominantly with the phosphate group of lipid heads (especially in the orientation 1). At the lower temperature, the choline and phosphate groups contribute approximately equally to the number of contacts between the protein and the membrane. Choline groups in blue, phosphate groups in yellow and oxygen atoms (glycerol backbone) in red. Top and bottom row represent data from 20 °C and 50 °C, while left and right columns represent orientation 1 and 2; Figure S8: Structure of MBP in different scenarios. (Left panel) at the endpoint of the orientation 1, *T* = 20 °C simulation, with MBP not being in contact with the lipid bilayer. (Middle panel) snapshot from orientation 2, *T* = 20 °C case, possessing most contacts/interactions with DPPC during its respective simulation. (Right panel) snapshot from orientation 2, *T* = 50 °C case, possessing most contacts/interactions with DPPC during its respective simulation. Phosphorous atoms belonging to the lipid headgroups are shown in transparent yellow, with the remainder of the bilayer given in gray. Protein regions interacting with the DPPC bilayer are shown in orange (residues 1 to 8), blue (residues 45 to 55), green (residues 136 to 144) and purple (residues 153 to 169); Figure S9: Principal component analysis was based on the Cα atoms of MBP backbone, with all structures projected onto the common (first two) principal components. In each case, structures representing the entire span of their respective simulations (*t* = 200 ns) were considered; Figure S10: Deuterium order parameter calculated for both *sn*1 and *sn*2 acyl chains of both DPPC leaflets (last 150 ns considered in all cases). Obtained order parameters show statistically insignificant difference to the reference pure DPPC systems at *T* = 20 °C and *T* = 50 °C (consult ref. [74] from the manuscript, SI, Figure S4); Table S1: Average number (#) of HBs and of contacts between MBP residues and the lipid bilayer in the last 150 ns of orientation 1 and orientation 2, *T* = 20 °C scenarios, respectively; Table S2: Average number (#) of HBs and of contacts between MBP residues and the lipid bilayer in the last 150 ns of orientation 1 and orientation 2, *T* = 50 °C scenarios, respectively.
