# Supplementary material for: Phase-Dependent Adsorption of Myelin Basic Protein to Phosphatidylcholine Lipid Bilayers
Source: Membranes (Basel). 2024 Jan 4;14(1):15. doi: 10.3390/membranes14010015 (PMC10819005; doi:10.3390/membranes14010015)
Supplement: Supplementary file 1 [file membranes-14-00015-s001.zip › membranes-2764625-supplementary.pdf]

Supporting Information

S1. DLS data and microscopic images of MBP, DPPC and MBP+DPPC p2

S2. DSC curves of MBP in $NaCl_{(aq)}$ p3

S3. Initial configurations of MBP+DPPC p4

S4. Additional CD data p5

S5. Additional molecular dynamics data p6

S1. DLS data and microscopic images of MBP, DPPC and MBP+DPPC

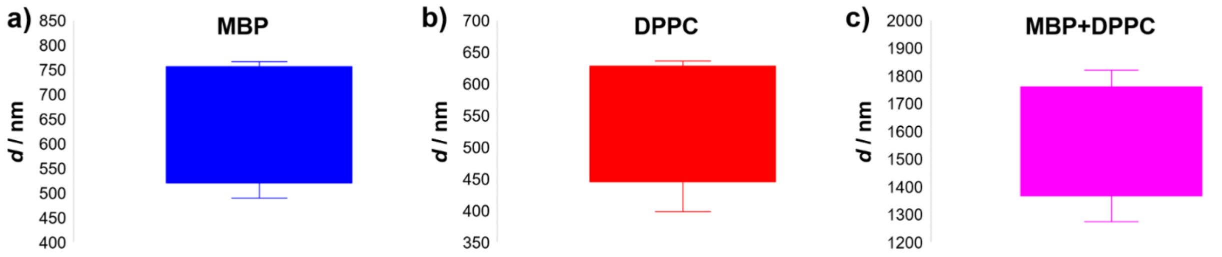

Figure S1. DLS data of: a) MBP; b) DPPC; c) MBP+DPPC in $NaCl_{(aq)}$ obtained at 25 °C.

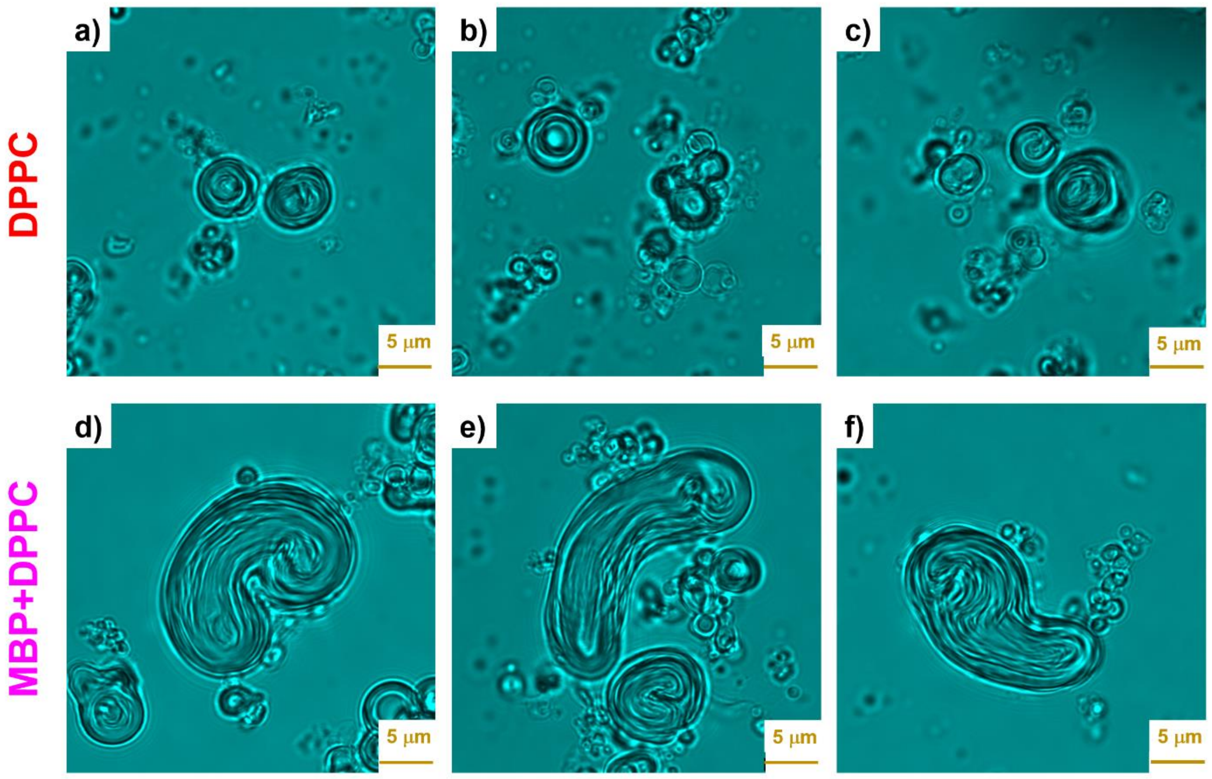

Figure S2. Confocal microscope images of: DPPC (upper row: a), b, c)) and MBP+DPPC (lower row: d), e), f)) in transmission.

S2. DSC curves of MBP in $NaCl_{(aq)}$

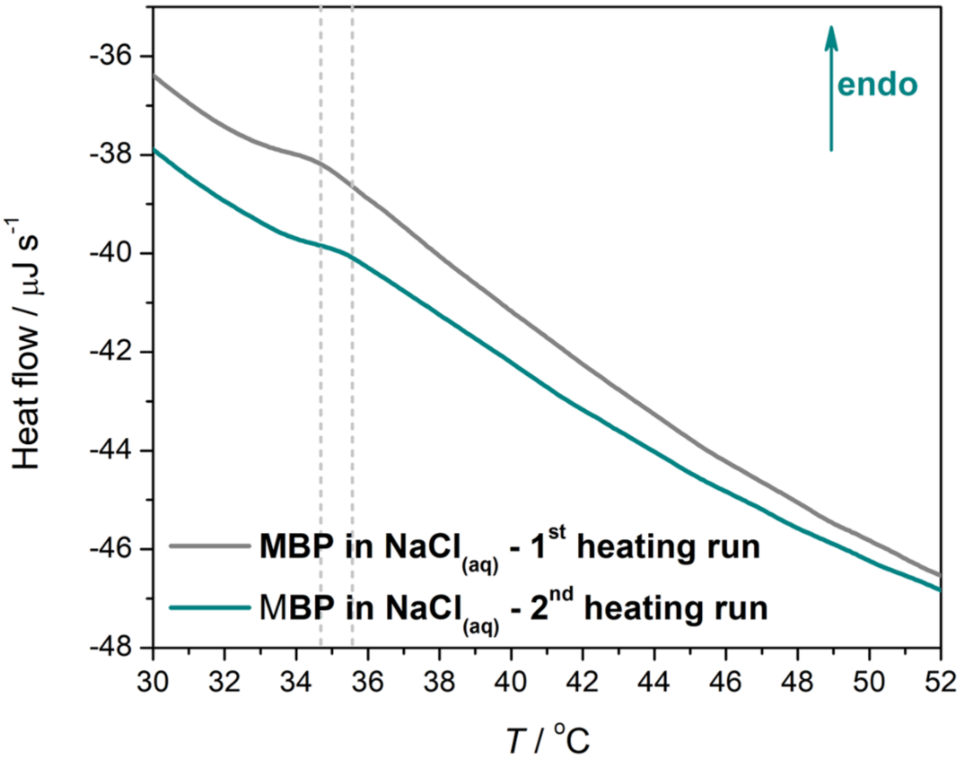

Figure S3. DSC curves (raw data) of MBP in $NaCl_{(aq)}$ obtained from the first (gray) and second (dark cyan) heating runs. Dashed lines designated estimated maximum of very weak thermotropic event.

S3. Initial configurations of MBP+DPPC

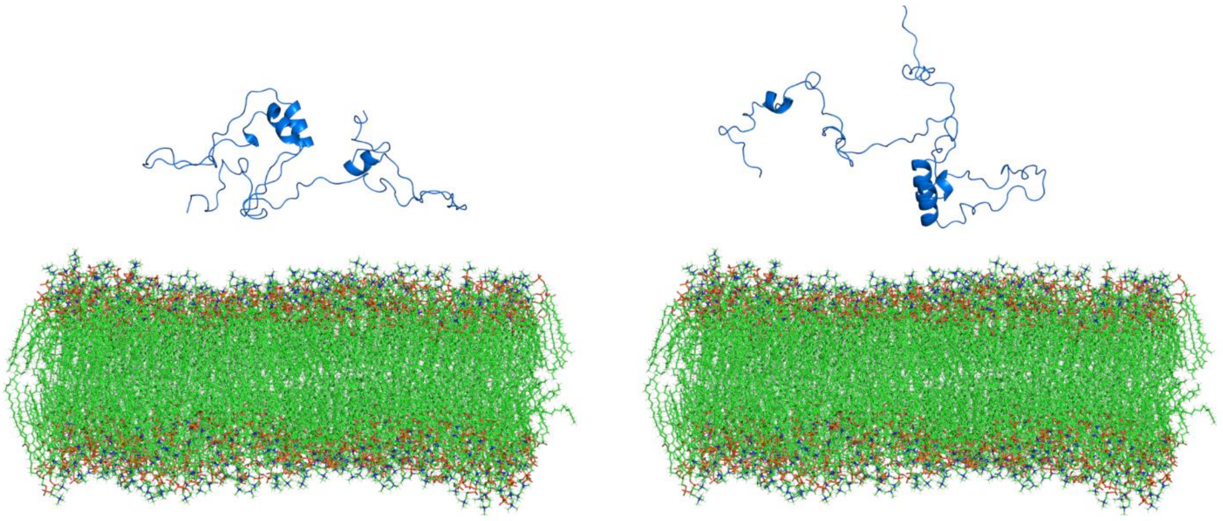

Fig S4. Equilibrated structures used as initial configurations for production simulations at 20 °C. MBP orientation 1 and orientation 2 (left and right, respectively).

S4. Additional CD data

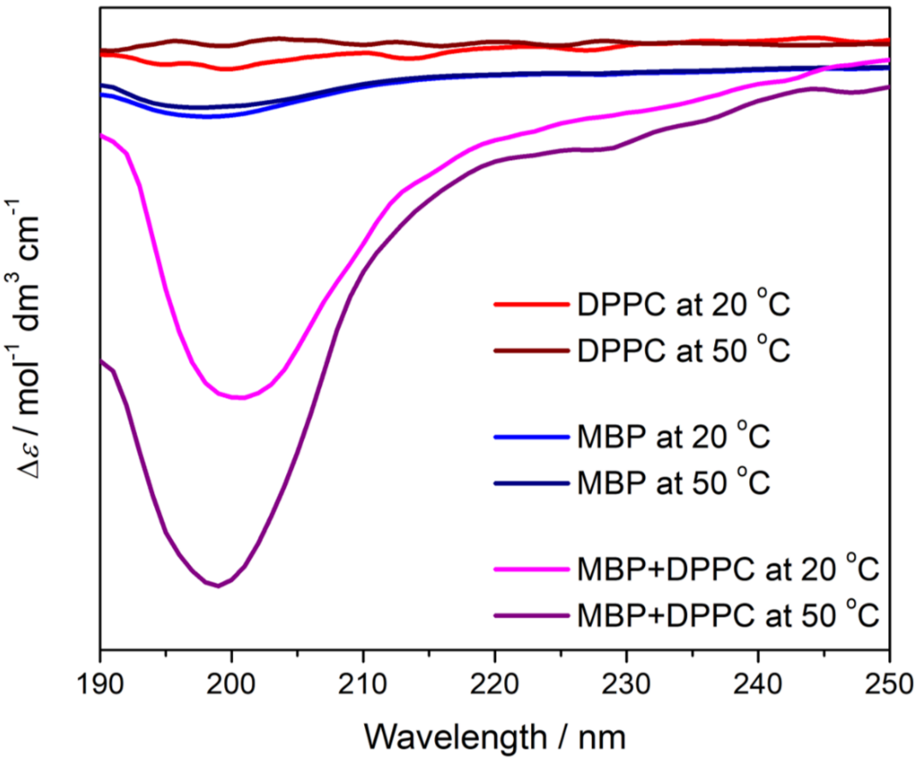

Fig S5. Mean smoothed CD spectra of MBP, DPPC and MBP+DPPC measured at 20 °C and 50 °C.

S5. Additional molecular dynamics data

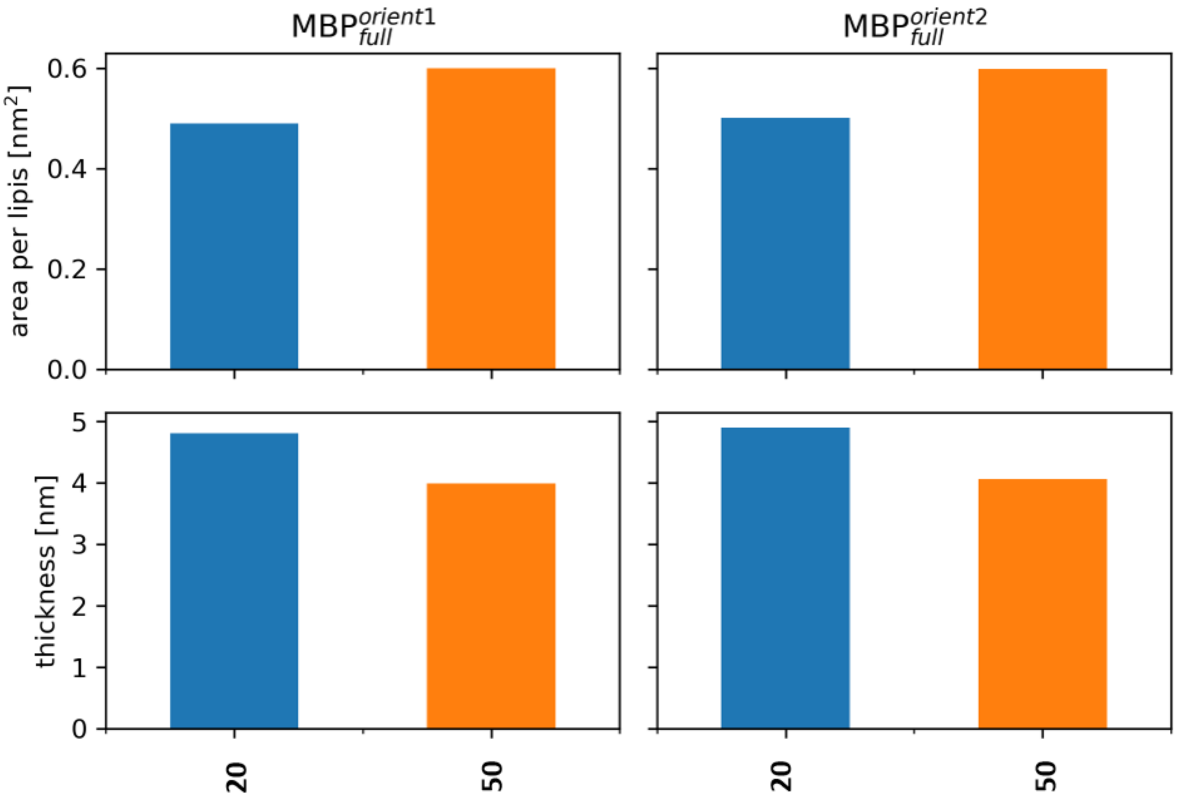

Fig S6. Membrane area per lipid (APL; nm^2) and thickness (nm) at 20 °C and 50 °C. As expected, with increased temperature, the area per lipid increases and thickness decreases. The properties are consistent in simulation of MBP in different initial orientations.

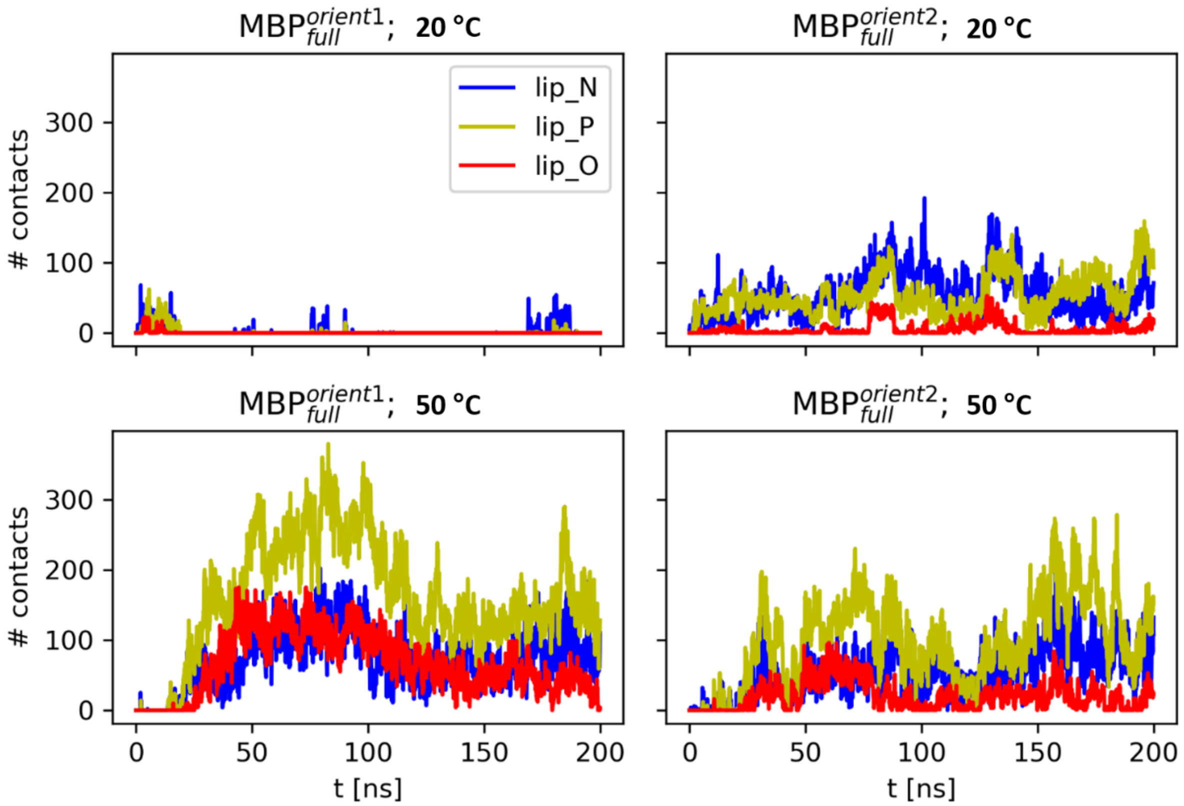

Fig S7. Type of interactions between MBP and DPPC. At higher temperature MBP forms contacts predominantly with the phosphate group of lipid heads (especially in the orientation 1). At the lower temperature, the choline and phosphate groups contribute approximately equally to the number of contacts between the protein and the membrane. Choline groups in blue, phosphate groups in yellow and oxygen atoms (glycerol backbone) in red. Top and bottom row represent data from 20 °C and 50 °C, while left and right columns represent orientation 1 and 2.

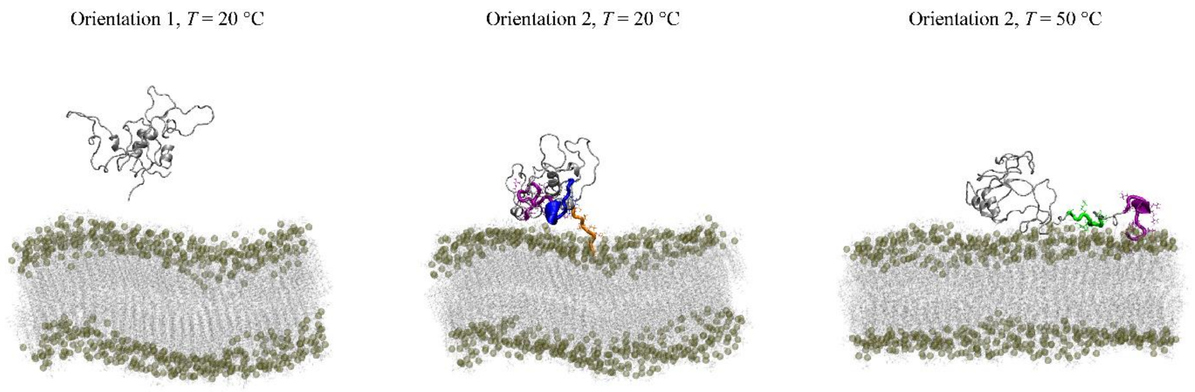

Fig S8. Structure of MBP in different scenarios. Left panel) at the endpoint of the orientation 1, T = 20 °C simulation, with MBP not being in contact with the lipid bilayer. Middle panel) snapshot from orientation 2, T = 20 °C case, possessing most contacts/interactions with DPPC during its respective simulation. Right panel) snapshot from orientation 2, T = 50 °C case, possessing most contacts/interactions with DPPC during its respective simulation. Phosphorous atoms belonging to the lipid headgroups are shown in transparent yellow, with the remainder of the bilayer given in gray. Protein regions interacting with the DPPC bilayer are shown in orange (residues 1 to 8), blue (residues 45 to 55), green (residues 136 to 144) and purple (residues 153 to 169).

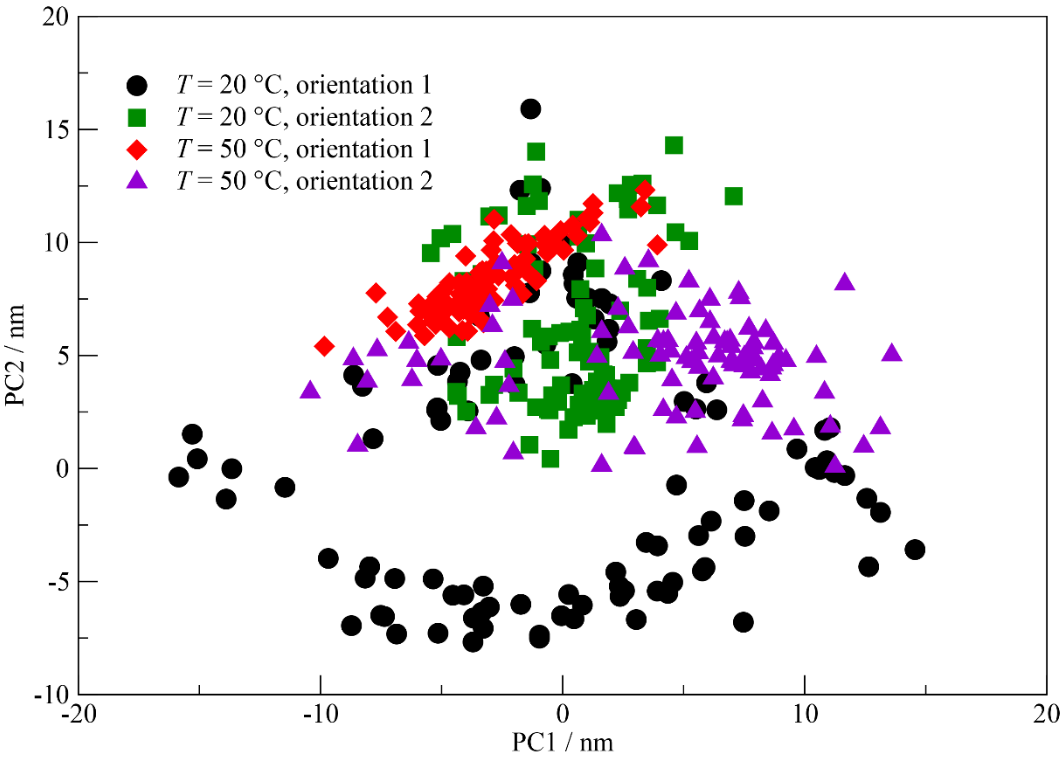

Fig S9. Principal component analysis was based on the Cα atoms of MBP backbone, with all structures projected onto the common (first two) principal components. In each case, structures representing the entire span of their respective simulations (t = 200 ns) were considered.

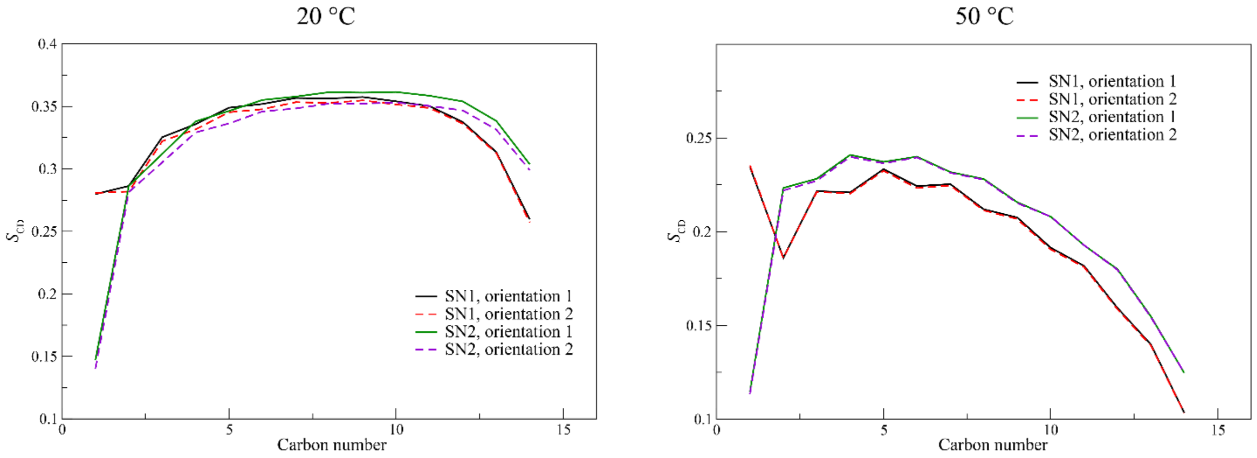

Fig S10. Deuterium order parameter calculated for both *sn1* and *sn2* acyl chains of both DPPC leaflets (last 150 ns considered in all cases). Obtained order parameters show statistically insignificant difference to the reference pure DPPC systems at *T* = 20 °C and *T* = 50 °C (consult ref. 74 from the manuscript, SI, Figure S4).

Table S1. Average number (#) of HBs and of contacts between MBP residues and the lipid bilayer in the last 150 ns of orientation 1 and orientation 2, T = 20 °C scenarios, respectively.

	**orientation 1**		**orientation 2**	
**Residue**	Average # of HBs	Average # of contacts	Average # of HBs	Average # of contacts
1ALA	0	0.00	0.09	24.55
2ALA	0	0.00	0.07	10.75
3GLN	0	0.00	0.00	13.67
4LYS	0	0.00	0.09	21.17
5ARG	0	0.00	0.00	2.09
6PRO	0	0.00	0.00	0.04
7SER	0	0.00	0.00	0.17
8GLN	0	0.00	0.00	0.00
9ARG	0	0.00	0.01	3.20
10SER	0	0.00	0.00	0.00
11LYS	0	0.00	0.00	0.00
12TYR	0	0.00	0.00	0.00
13LEU	0	0.00	0.00	0.00
14ALA	0	0.00	0.00	0.00
15SER	0	0.00	0.00	0.00
16ALA	0	0.00	0.00	0.00
17SER	0	0.00	0.00	0.00
18THR	0	0.00	0.00	0.00
19MET	0	0.00	0.00	0.00
20ASP	0	0.00	0.00	0.00
21HSD	0	0.00	0.00	0.00
22ALA	0	0.00	0.00	0.00
23ARG	0	0.00	0.00	0.00
24HSD	0	0.00	0.00	0.00
25GLY	0	0.00	0.00	0.00
26PHE	0	0.00	0.00	0.00
27LEU	0	0.00	0.00	0.00
28PRO	0	0.00	0.00	0.00
29ARG	0	0.00	0.00	0.00

	orientation 1		orientation 2	
**Residue**	Average # of HBs	Average # of contacts	Average # of HBs	Average # of contacts
30HSD	0	0.00	0.00	0.00
31ARG	0	0.00	0.00	0.00
32ASP	0	0.00	0.00	0.00
33THR	0	0.00	0.00	0.00
34GLY	0	0.00	0.00	0.00
35ILE	0	0.00	0.00	0.00
36LEU	0	0.00	0.00	0.99
37ASP	0	0.00	0.00	0.00
38SER	0	0.00	0.00	0.00
39LEU	0	0.00	0.00	0.01
40GLY	0	0.00	0.00	0.00
41ARG	0	0.00	0.00	0.00
42PHE	0	0.00	0.00	0.00
43PHE	0	0.00	0.00	2.76
44GLY	0	0.00	0.00	0.14
45SER	0	0.00	0.00	10.96
46ASP	0	0.00	0.00	19.62
47ARG	0	0.00	0.00	21.50
48GLY	0	0.00	0.00	30.42
49ALA	0	0.00	0.00	9.07
50PRO	0	0.00	0.00	29.03
51LYS	0	0.00	0.72	92.12
52ARG	0	0.00	0.00	2.21
53GLY	0	0.00	0.00	2.25
54SER	0	0.00	0.00	0.01
55GLY	0	0.00	0.00	0.00
56LYS	0	0.00	0.00	0.00
57ASP	0	0.00	0.00	0.00
58GLY	0	0.00	0.00	0.00
59HSD	0	0.00	0.00	8.49
60HSD	0	0.00	0.00	24.70
61ALA	0	0.00	0.00	10.84

	orientation 1		orientation 2	
**Residue**	Average # of HBs	Average # of contacts	Average # of HBs	Average # of contacts
62ALA	0	0.00	0.00	9.93
63ARG	0	0.00	0.00	4.75
64THR	0	0.00	0.00	0.71
65THR	0	0.05	0.00	1.74
66HSD	0	2.24	0.00	2.36
67TYR	0	6.20	0.00	0.13
68GLY	0	1.17	0.00	0.00
69SER	0	2.62	0.00	0.00
70LEU	0	0.18	0.00	0.00
71PRO	0	0.22	0.00	0.00
72GLN	0	0.00	0.00	0.00
73LYS	0	0.00	0.00	0.00
74ALA	0	0.00	0.00	0.00
75GLN	0	0.00	0.00	0.00
76GLY	0	0.00	0.00	0.00
77HSD	0	0.00	0.00	0.00
78ARG	0	0.00	0.00	0.00
79PRO	0	0.00	0.00	0.00
80GLN	0	0.00	0.00	0.00
81ASP	0	0.00	0.00	0.00
82GLU	0	0.00	0.00	0.00
83ASN	0	0.00	0.00	0.00
84PRO	0	0.00	0.00	0.00
85VAL	0	0.00	0.00	0.00
86VAL	0	0.00	0.00	0.00
87HSD	0	0.00	0.00	0.00
88PHE	0	0.00	0.00	0.00
89PHE	0	0.00	0.00	0.00
90LYS	0	0.00	0.00	0.00
91ASN	0	0.00	0.00	0.00
92ILE	0	0.00	0.00	0.00
93VAL	0	0.00	0.00	0.00

	orientation 1		orientation 2	
**Residue**	Average # of HBs	Average # of contacts	Average # of HBs	Average # of contacts
94THR	0	0.00	0.00	0.00
95PRO	0	0.00	0.00	0.00
96ARG	0	0.00	0.00	0.00
97THR	0	0.00	0.00	0.00
98PRO	0	0.00	0.00	0.00
99PRO	0	0.00	0.00	0.00
100PRO	0	0.00	0.00	0.00
101SER	0	0.00	0.00	0.00
102GLN	0	0.00	0.00	0.00
103GLY	0	0.00	0.00	0.00
104LYS	0	0.00	0.00	0.00
105GLY	0	0.00	0.00	0.00
106ARG	0	0.00	0.00	0.00
107GLY	0	0.00	0.00	0.00
108LEU	0	0.00	0.00	0.00
109SER	0	0.00	0.00	0.00
110LEU	0	0.00	0.00	0.00
111SER	0	0.00	0.00	0.00
112ARG	0	0.00	0.00	0.00
113PHE	0	0.00	0.00	0.00
114SER	0	0.00	0.00	0.00
115TRP	0	0.00	0.00	0.00
116GLY	0	0.00	0.00	0.00
117ALA	0	0.00	0.00	0.00
118GLU	0	0.00	0.00	0.00
119GLY	0	0.00	0.00	0.00
120GLN	0	0.00	0.00	1.16
121LYS	0	0.00	0.00	0.01
122PRO	0	0.00	0.00	2.37
123GLY	0	0.00	0.00	4.45
124PHE	0	0.00	0.00	7.95
125GLY	0	0.00	0.00	18.18

	orientation 1		orientation 2	
**Residue**	Average # of HBs	Average # of contacts	Average # of HBs	Average # of contacts
126TYR	0	0.00	0.00	23.42
127GLY	0	0.00	0.00	29.86
128GLY	0	0.00	0.09	45.14
129ARG	0	0.00	1.04	177.07
130ALA	0	0.00	0.00	6.37
131SER	0	0.00	0.07	14.86
132ASP	0	0.00	0.00	6.21
133TYR	0	0.00	0.00	0.08
134LYS	0	0.00	0.08	14.26
135SER	0	0.00	0.00	0.28
136ALA	0	0.00	0.00	0.00
137HSD	0	0.00	0.00	0.09
138LYS	0	0.00	0.00	0.00
139GLY	0	0.00	0.00	0.00
140LEU	0	0.00	0.00	0.00
141LYS	0	0.00	0.00	0.00
142GLY	0	0.00	0.00	0.00
143HSD	0	0.00	0.00	0.00
144ASP	0	0.00	0.00	0.00
145ALA	0	0.00	0.00	0.00
146GLN	0	0.00	0.00	0.00
147GLY	0	0.00	0.00	0.00
148THR	0	0.00	0.00	0.26
149LEU	0	0.00	0.00	0.62
150SER	0	0.00	0.00	0.00
151LYS	0	0.00	0.00	0.00
152ILE	0	0.00	0.00	14.26
153PHE	0	0.00	0.00	22.96
154LYS	0	0.00	0.04	10.64
155LEU	0	0.00	0.00	0.93
156GLY	0	0.00	0.00	0.05
157GLY	0	0.00	0.00	0.00

	orientation 1		orientation 2	
**Residue**	Average # of HBs	Average # of contacts	Average # of HBs	Average # of contacts
158ARG	0	0.00	0.00	0.04
159ASP	0	0.00	0.00	0.00
160SER	0	0.00	0.00	0.00
161ARG	0	0.00	0.00	0.00
162SER	0	0.00	0.00	0.04
163GLY	0	0.00	0.00	0.00
164SER	0	0.00	0.00	0.47
165PRO	0	0.00	0.00	0.70
166MET	0	0.00	0.00	0.91
167ALA	0	0.00	0.00	0.12
168ARG	0	0.00	0.05	5.82
169ARG	0	0.00	0.07	15.61

Table S2. Average number (#) of HBs and of contacts between MBP residues and the lipid bilayer in the last 150 ns of orientation 1 and orientation 2, T = 50 °C scenarios, respectively.

	**orientation 1**		**orientation 2**	
**Residue**	Average # of HBs	Average # of contacts	Average # of HBs	Average # of contacts
1ALA	0.03	5.71	0.01	1.36
2ALA	0.00	5.09	0.00	0.00
3GLN	0.00	5.33	0.00	0.00
4LYS	0.16	31.09	0.00	0.00
5ARG	0.37	30.88	0.00	0.00
6PRO	0.00	5.16	0.00	0.00
7SER	0.00	0.09	0.00	0.00
8GLN	0.00	7.28	0.00	0.00
9ARG	0.00	0.00	0.00	0.00
10SER	0.00	0.00	0.00	0.00
11LYS	0.00	0.14	0.00	0.00
12TYR	0.00	0.00	0.00	0.00
13LEU	0.00	0.00	0.00	0.00
14ALA	0.00	0.00	0.00	0.00
15SER	0.00	1.01	0.00	0.00
16ALA	0.00	1.20	0.00	0.00
17SER	0.00	0.58	0.00	0.00
18THR	0.00	6.70	0.00	0.00
19MET	0.00	3.32	0.00	0.00
20ASP	0.00	3.71	0.00	0.00
21HSD	0.00	2.83	0.00	0.00
22ALA	0.00	1.04	0.00	0.00
23ARG	0.05	6.18	0.00	0.00
24HSD	0.00	0.00	0.00	0.00
25GLY	0.00	0.00	0.00	0.00
26PHE	0.00	0.20	0.00	0.00
27LEU	0.00	0.00	0.00	0.00
28PRO	0.00	0.00	0.00	0.00

	orientation 1		orientation 2	
Residue	Average # of HBs	Average # of contacts	Average # of HBs	Average # of contacts
29ARG	0.00	0.25	0.00	0.00
30HSD	0.00	0.00	0.00	0.00
31ARG	0.00	1.20	0.00	0.00
32ASP	0.00	0.07	0.00	0.00
33THR	0.00	0.00	0.00	0.05
34GLY	0.00	0.00	0.00	0.00
35ILE	0.00	0.00	0.00	0.01
36LEU	0.00	0.03	0.00	0.00
37ASP	0.00	0.46	0.00	0.00
38SER	0.00	0.00	0.00	0.00
39LEU	0.00	0.00	0.00	0.00
40GLY	0.00	0.29	0.00	0.00
41ARG	0.00	0.38	0.00	0.00
42PHE	0.00	0.04	0.00	0.00
43PHE	0.00	4.03	0.00	0.00
44GLY	0.00	10.34	0.00	0.00
45SER	0.00	24.11	0.00	0.00
46ASP	0.00	12.37	0.00	0.00
47ARG	0.75	125.76	0.00	0.00
48GLY	0.01	7.59	0.00	0.00
49ALA	0.00	3.36	0.00	0.00
50PRO	0.00	4.22	0.00	0.00
51LYS	0.09	24.38	0.00	0.00
52ARG	0.20	27.09	0.00	0.00
53GLY	0.00	2.95	0.00	0.00
54SER	0.01	4.92	0.00	0.00
55GLY	0.00	0.62	0.00	0.01
56LYS	0.00	0.83	0.01	1.14
57ASP	0.00	2.08	0.00	0.00
58GLY	0.00	0.05	0.00	0.00
59HSD	0.00	0.79	0.00	0.00

	orientation 1		orientation 2	
Residue	Average # of HBs	Average # of contacts	Average # of HBs	Average # of contacts
60HSD	0.00	0.24	0.00	0.00
61ALA	0.00	0.00	0.00	0.00
62ALA	0.00	0.00	0.00	0.00
63ARG	0.00	0.00	0.00	0.00
64THR	0.00	0.00	0.00	0.00
65THR	0.00	0.00	0.00	0.00
66HSD	0.00	0.00	0.00	0.00
67TYR	0.00	0.00	0.00	0.00
68GLY	0.00	0.00	0.00	0.00
69SER	0.00	0.00	0.00	0.00
70LEU	0.00	0.00	0.00	0.00
71PRO	0.00	0.00	0.00	0.00
72GLN	0.00	0.00	0.00	0.00
73LYS	0.00	0.00	0.00	0.00
74ALA	0.00	0.00	0.00	0.00
75GLN	0.00	0.00	0.00	0.01
76GLY	0.00	0.00	0.00	0.00
77HSD	0.00	0.00	0.00	1.64
78ARG	0.07	4.93	0.01	3.30
79PRO	0.00	0.01	0.00	0.00
80GLN	0.01	3.74	0.00	0.11
81ASP	0.00	2.42	0.00	0.00
82GLU	0.00	0.00	0.00	0.00
83ASN	0.00	0.28	0.00	0.00
84PRO	0.00	0.22	0.00	0.00
85VAL	0.00	0.00	0.00	0.00
86VAL	0.00	0.00	0.00	0.00
87HSD	0.00	0.00	0.00	0.00
88PHE	0.00	0.00	0.00	0.00
89PHE	0.00	0.00	0.00	0.01
90LYS	0.00	0.00	0.00	0.04

	orientation 1		orientation 2	
**Residue**	Average # of HBs	Average # of contacts	Average # of HBs	Average # of contacts
91ASN	0.00	0.00	0.00	0.00
92ILE	0.00	0.00	0.00	0.00
93VAL	0.00	0.00	0.00	0.78
94THR	0.00	0.00	0.00	0.18
95PRO	0.00	0.00	0.00	2.70
96ARG	0.00	0.00	0.00	0.34
97THR	0.00	0.00	0.00	0.08
98PRO	0.00	0.00	0.00	2.16
99PRO	0.00	0.00	0.00	0.41
100PRO	0.00	0.00	0.00	0.00
101SER	0.00	0.00	0.00	3.32
102GLN	0.00	0.00	0.00	9.11
103GLY	0.00	0.00	0.00	7.47
104LYS	0.00	0.00	0.43	50.33
105GLY	0.00	0.00	0.00	3.29
106ARG	0.00	0.00	0.00	1.61
107GLY	0.00	0.00	0.00	2.76
108LEU	0.00	0.00	0.00	37.17
109SER	0.00	0.00	0.22	25.63
110LEU	0.00	0.00	0.00	4.30
111SER	0.00	0.00	0.00	3.80
112ARG	0.00	0.00	0.67	62.37
113PHE	0.00	0.00	0.00	16.43
114SER	0.00	0.00	0.00	0.18
115TRP	0.00	0.00	0.00	5.49
116GLY	0.00	0.00	0.00	0.47
117ALA	0.00	0.00	0.00	0.04
118GLU	0.00	0.00	0.00	0.00
119GLY	0.00	0.00	0.00	0.00
120GLN	0.00	0.00	0.00	0.00
121LYS	0.00	0.00	0.00	0.00

	orientation 1		orientation 2	
**Residue**	Average # of HBs	Average # of contacts	Average # of HBs	Average # of contacts
122PRO	0.00	0.00	0.00	0.00
123GLY	0.00	0.00	0.00	0.01
124PHE	0.00	0.00	0.00	1.29
125GLY	0.00	0.00	0.00	4.74
126TYR	0.00	0.00	0.01	23.16
127GLY	0.00	0.00	0.00	27.11
128GLY	0.00	0.00	0.13	44.36
129ARG	0.00	0.00	1.91	202.82
130ALA	0.00	0.00	0.01	11.05
131SER	0.00	0.00	0.11	42.26
132ASP	0.00	0.00	0.00	14.22
133TYR	0.00	0.00	0.05	28.92
134LYS	0.00	0.05	0.38	53.95
135SER	0.00	0.00	0.08	21.25
136ALA	0.00	0.87	0.01	11.64
137HSD	0.01	8.80	0.00	10.07
138LYS	0.07	22.95	0.16	26.82
139GLY	0.00	4.30	0.00	2.83
140LEU	0.00	10.26	0.00	13.92
141LYS	0.34	59.21	0.07	16.21
142GLY	0.00	29.05	0.00	0.78
143HSD	0.08	51.80	0.00	3.62
144ASP	0.00	6.01	0.00	0.62
145ALA	0.00	4.78	0.00	1.50
146GLN	0.00	4.71	0.00	2.29
147GLY	0.00	0.00	0.00	0.47
148THR	0.00	0.00	0.00	2.62
149LEU	0.00	0.00	0.00	1.67
150SER	0.00	0.01	0.00	0.74
151LYS	0.00	1.01	0.00	0.80
152ILE	0.00	0.00	0.00	1.07

	orientation 1		orientation 2	
**Residue**	Average # of HBs	Average # of contacts	Average # of HBs	Average # of contacts
153PHE	0.00	0.00	0.00	3.61
154LYS	0.00	5.54	0.03	8.20
155LEU	0.00	2.50	0.00	3.78
156GLY	0.00	6.50	0.01	1.54
157GLY	0.03	3.67	0.00	0.84
158ARG	2.66	247.45	0.09	12.43
159ASP	0.51	143.76	0.00	5.89
160SER	0.82	211.05	0.00	3.16
161ARG	2.20	329.33	0.30	27.74
162SER	0.97	93.25	0.00	11.32
163GLY	0.11	6.86	0.05	10.66
164SER	0.00	0.03	0.07	20.93
165PRO	0.00	3.66	0.00	19.09
166MET	0.00	0.68	0.00	21.95
167ALA	0.00	0.16	0.11	33.50
168ARG	0.53	81.68	0.91	122.91
169ARG	0.78	127.90	1.07	113.36
